# Preparation of aminoethyl glycosides for glycoconjugation

**DOI:** 10.3762/bjoc.6.81

**Published:** 2010-07-29

**Authors:** Robert Šardzík, Gavin T Noble, Martin J Weissenborn, Andrew Martin, Simon J Webb, Sabine L Flitsch

**Affiliations:** 1 Manchester Interdisciplinary Biocentre & School of Chemistry, The University of Manchester, 131 Princess Street, Manchester, M1 7DN, UK

**Keywords:** aminoethyl glycosides, glycoarrays, glycoconjugation, glycosylation

## Abstract

The synthesis of a number of aminoethyl glycosides of cell-surface carbohydrates, which are important intermediates for glycoarray synthesis, is described. A set of protocols was developed which provide these intermediates, in a short number of steps, from commercially available starting materials.

## Introduction

The chemical conjugation of carbohydrates through the anomeric centre to biomolecules such as peptides, proteins, lipids, metabolites and to array surfaces is an important synthetic challenge [1–5]. A diverse range of linkers and spacers has been described in the literature [2–12], among which aminoalkyl glycosides have become the most popular, in particular the aminoethyl linker. This linker has been tested in a large number of arrays and appears to be biocompatible in array screening [2–3,7]. Given that aminoethyl glycosides are conveniently conjugated to surfaces containing activated carboxylates, they have become a useful generic anomeric functional group for glycoconjugation. The importance of this linker merits efforts into finding a robust synthetic method than can be used by scientists who are not experienced in carbohydrate synthesis. Here we describe a systematic study with the aim of finding such robust and efficient methods for a number of commonly used mono- and disaccharides starting from commercially available reagents and with a minimal number of steps. In our studies no such general aminoethylation method that was applicable to all targets was found, which rather suggests that protocols need to be tailored for each sugar.

## Results and Discussion

### Coupling reactions

Aminoethyl glycosides have previously been generated in a number of ways. Free sugars have been glycosylated with 2-chloroethanol under acid catalysis, followed by peracetylation, nucleophilic substitution with azide and finally, reduction of the azido group [13–14]. Alternatively, the carbohydrate was first activated as the trichloroacetimidate or bromide followed by glycosylation with *N*-Cbz-aminoethanol [15], bromoethanol [16] or azidoethanol [17] and subsequently transformed into the amine.

**Figure 1:**
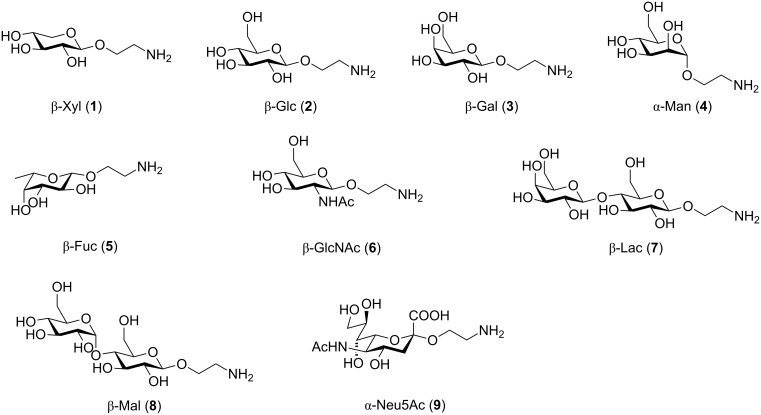
Aminoethyl glycosides (**1**–**9**) which were synthesised in this study.

**Scheme 1:**
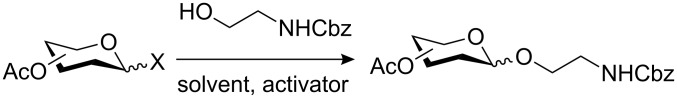
General reaction scheme for generation of aminoethyl glycosides. X = OAc, Br or Cl.

In the interest of finding fast reliable methods, we have investigated two general aminoethylation protocols: First, the direct glycosylation of peracetylated sugars, which can be either purchased or easily prepared from free sugars and can be used without purification. Where these proved to be unreactive, the anomeric acetates were converted to glycosyl bromides, usually in quantitative yields, and products were used without further purification. Where possible, *N*-Cbz-protected aminoethanol was used as the glycosyl acceptor because it is commercially available, crystalline and can be easily deprotected in one step avoiding use of azides. Figure 1 lists the target aminoethyl glycosides (**1**–**9**) generated in this study (q.v. Scheme 1 and Table 1).

The key glycosylation step is shown in Scheme 1 and the results of the different glycosylation reactions are summarised in Table 1.

**Table 1:** Results of glycosylation reactions as shown in Scheme 1.

Entry	Product	X	Method	α/β^c^	Yield^d^

1	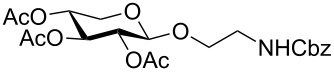 **10**	Br	A	17:83	67 + 23^e^
2	**10**	Br	B	23:77	58 + 19^e^
3	**10**	OAc	C	22:78	52 + 17^e^
4	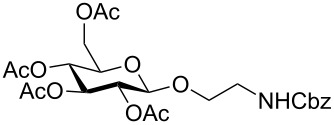 **11**	OAc	C	15:85	36
5	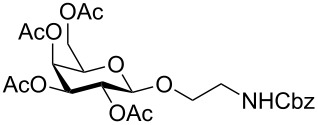 **12**	OAc	C	8:92	62
6	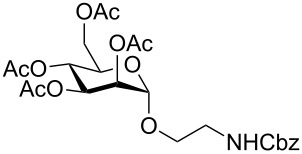 **13**	OAc	C	>95:5	57
7	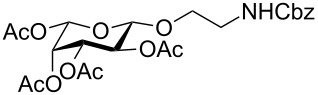 **14**	Br	A	14:86	75 + 19^e^
8	**14**	Br	B	15:85	75 + 20^e^
9	**14**	OAc	C	35:65	n.d.
10	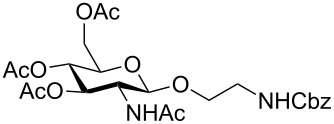 **15**	OAc	D	>5:95	61
11	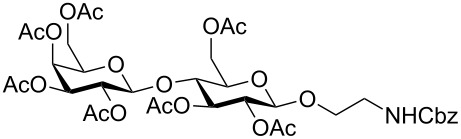 **16**	Br	A	10:90	56
12	**16**	Br	B	10:90	86
13	**16**	Br	A^a^	31:68	30
14	**16**	Br	B^b^	30:70	73^e^
15	**16**	Br	E	37:63	59
16	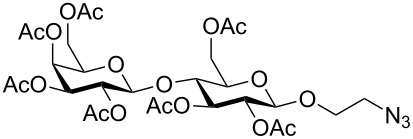 **17**	Br	E	10:90	88
17	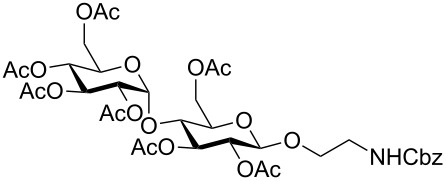 **18**	Br	B	14:86	47
18	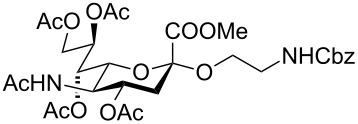 **19**	Cl	F	10:90	70

^a^The reaction was performed overnight at r.t. ^b^The reaction was performed in CH_2_Cl_2_. ^c^determined by ^13^C NMR from crude reaction mixture. ^d^Yield of pure major anomer (>95:5) after column chromatography. ^e^Mixture of both anomers. Method A: Hg(CN)_2_, CH_3_CN, 60 °C, 2–4 h. Method B: Hg(CN)_2_, CH_3_CN, 90 °C, microwave, 200 W, 15 min. Method C: BF_3_·Et_2_O, CH_3_CN, 0 °C, 1 h, r.t., overnight. Method D: SnCl_4_, CH_3_CN, 60 °C, 16 h. Method E: Hg(CN)_2_, HgBr_2_, CH_3_CN, r.t., overnight. Method F: Ag_2_CO_3_, CH_2_Cl_2_, r.t., overnight.

Fully protected xylopyranoside **10** could be prepared both from the corresponding bromide as well as the acetate (the β-anomer was prepared from xylose with sodium acetate in acetic anhydride) in similar yields. In each case both anomers were formed with moderate selectivity and from the reaction mixture the pure β-anomer (>95:5) was isolated by column chromatography.

The glucoside **11**, galactoside **12** and mannoside **13** were prepared in moderate to good yield directly from the acetate (X = OAc in Scheme 1) giving rapid access to these monosaccharide derivatives.

The fucoside **14** was generated both from the acetate and bromide. Higher alpha selectivity was observed with the bromide (α/β ratio of crude product 82:18).

*N*-Acetylglucosamine **15** was successfully prepared from the β-acetate using SnCl_4_ (method D). When starting from the α-acetate, no reaction was observed and only starting material could be recovered. Under microwave conditions (method B or using other Lewis acids as Yb(OTf)_3_ in DCM, 90 °C, 30 min, 200 W) the reaction was not reproducible, giving low yields and leading to decomposition products.

Lactose is both cheap and readily available. It is an important component of glycoprotein glycans and also a substrate for sialyltransferases to generate biologically important sialyllactosides. The aminoethyl lactoside **16** was prepared in greatest yield from the bromide and attempts to prepare **16** directly from the acetate using BF_3_·Et_2_O as the activator only resulted in decomposition.

A number of reaction conditions for generating **16** from the bromide with *N*-Cbz-aminoethanol were investigated. With Ag_2_CO_3_ in dichloromethane at room temperature low yields of product **16** along with a number of side-products (orthoester, elimination or hydrolysis) were observed and the product was difficult to separate from starting materials, in particular the *N*-Cbz-aminoethanol. With Hg(CN)_2_, or the more reactive Hg(CN)_2_/HgBr_2_-mixture, in dichloromethane or acetonitrile, glycosylation was more successful, but both anomers were generated. Given the problems previously encountered with purification, the glycosylation with Hg(CN)_2_ was further optimised by increasing both the temperature and the amount of acceptor. The problem of separation of the alcohol from the product was solved by acetylation of the crude reaction mixture to lower the polarity of the free alcohol. Attempts to speed up the reaction by heating led to the observation that in acetonitrile predominantly one (β) anomer is formed, but anomerisation occurs with longer reaction times. In dichloromethane both anomers were formed. The best reaction conditions were combined to give Method A. The success of Method A led to attempts to improve the method further and to use microwave irradiation as in method B. Method B also works well for monosaccharides and maltoside.

Given the problems with purification, the use of azidoethanol as a glycosyl acceptor was also investigated. This reaction (Table 1) was much more successful and produced mainly the beta anomer **17**.

Maltoside **18** was generated by the same microwave-mediated glycosylation as developed for lactoside **16** (Method B) and in reasonable yield.

*N*-Acetyl neuraminic acid (sialic acid) is an important component of cell surfaces and chemical glycosylation procedures involving sialic acid are generally challenging. In our hands activation as the chloride (prepared from Neu5Ac in 3 steps) using silver carbonate (Method F) gave reasonable yields of **19**.

### Deprotection reactions

**Scheme 2:**
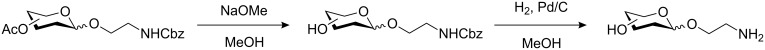
Deprotection protocols.

The general deprotection for compounds **10**–**18** is shown in Scheme 2. Acetates were cleaved using NaOMe followed by hydrogenation to generate **1**–**8** in good yields.

Deprotection was also successful when the hydrogenation was performed first, but in some cases migration of acetate to the aminoethyl linker was observed. However, this can be avoided by using palladium hydroxide on charcoal as the hydrogenation catalyst, with short reaction times, followed by the immediate use of the resulting amine in further coupling [18].

Sialoside **19** was deprotected by treatment with NaOMe, followed by LiOH and subsequent hydrogenation to give **9**.

## Conclusion

We have described rapid and convenient methods for the synthesis of a range of aminoethyl glycosides (**1**–**9**) of common mono- and disaccharides. Although some of the glycosylation reactions could be improved by using alternative glycosylation methods (such as trichloroacetimidates, thiols), these would require more steps with chromatographic purifications and less overall yields. These aminoethyl glycosides are now readily accessible for incorporation into glycan arrays.

## Supporting Information

A Supporting Information containing all experimental details and analytical data of all compounds described in the article as well as their precursors is available.

File 1 Experimental procedures and analytical data
