# Supplementary material for: Preparation of aminoethyl glycosides for glycoconjugation
Source: Beilstein J Org Chem. 2010 Jul 29;6:699–703. doi: 10.3762/bjoc.6.81 (PMC2956404; doi:10.3762/bjoc.6.81)
Supplement: File 1 — Experimental procedures and analytical data [file Beilstein_J_Org_Chem-06-699-s001.pdf]

Supporting Information

for

Preparation of aminoethyl glycosides for glycoconjugation

Robert Šardzík, Gavin T. Noble, Martin J. Weissenborn, Andrew Martin, Simon Webb and Sabine L. Flitsch*

Address: Manchester Interdisciplinary Biocentre & School of Chemistry, The University of Manchester, 131 Princess Street, Manchester, M1 7DN, UK

Email: Sabine L. Flitsch* - Sabine.Flitsch@manchester.ac.uk; Robert Šardzík - Robert.Sardzik@manchester.ac.uk

* Corresponding author

Experimental procedures and analytical data

General Methods

Unless stated otherwise, all chemicals were of analytical grade and used as received from Sigma-Aldrich. All solvents used were from commercial suppliers (Sigma-Aldrich, Fisher Scientific or Romil). Microwave mediated reactions were performed on an CEM-Discover SP-D workstation fitted with an automated Explorer 24/48 module in pressurised vessels (10 or 35 mL) with snap-on caps. NMR spectra were recorded on Bruker Avance 300, DPX400 and Avance II+ 500 spectrometers at room temperature and calibrated according to the chemical shift of tetramethysilane or 3-(trimethylsilyl)propionic-*2,2,3,3*-d_4 acid sodium salt for samples in D_2O (δ = 0 ppm). Compound spectra were assigned with ^1H, ^{13}C, DEPT, COSY, HSQC, HMQC and HMBC NMR experiments as appropriate. Chemical shifts are given in ppm, coupling constants in Hertz (Hz) and multiplicities indicated with the appropriate abbreviations: singlet (s), doublet (d), triplet (t), double doublet (dd), double double doublet (ddd) and multiplet (m). The determination of diastereomeric ratios is based on comparison of signal intensities of separated signal pairs in ^{13}C NMR spectra. ES+ mass spectra were obtained with Micromass Prospec and Micromass Platform spectrometers. IR spectra were measured and recorded using a Perkin Elmer Spectrum RX I FT-IR Spectrometer. Optical activity was measured using an Optical Activity Ltd AA-1C00 polarimeter. Melting points were measured with a Gallenkamp apparatus and are not corrected.

General procedures

General Procedure 1; Glycosylation Method A

Glycosyl bromide and *N*-Cbz-aminoethanol (2 equiv) were dissolved in abs. CH_3CN under a nitrogen atmosphere. $Hg(CN)_2$ (1.1 equiv) was added and the reaction mixture heated at 70 °C for 2–4 h. After this time, the solvent was evaporated *in vacuo,* the residue redissolved in CH_2Cl_2, washed successively with water, $NaHCO_3$-solution and brine, dried over $MgSO_4$ and concentrated *in vacuo*. The crude product was purified by column chromatography on silica.

General Procedure 2; Glycosylation Method B

To a solution of the glycosyl bromide and *N*-Cbz-aminoethanol (2 equiv) in abs. CH_3CN in a microwave vessel was added $Hg(CN)_2$ (1.1 equiv), the vessel was closed and the reaction mixture heated at 90 °C for 15 min at 200 W power. The solvent was removed under vacuum, the residue redissolved in CH_2Cl_2, washed successively with water, $NaHCO_3$-solution and brine, dried over $MgSO_4$ and concentrated *in vacuo*. The crude product was purified by column chromatography on silica.

General Procedure 3; Glycosylation Method C

The peracetylated glycosyl acceptor (1 equiv) and *N*-Cbz-ethanolamine (1.2 equiv) were dissolved in dry acetonitrile under a nitrogen atmosphere. The solution was cooled to 0 °C and $BF_3 \cdot Et_2O$ (5 equiv) added dropwise. The reaction was stirred 30 min at 0 °C and then overnight at r.t. The reaction was quenched with Et_3N and concentrated *in vacuo*, the residue redissolved in dichloromethane then washed successively with sat. $NaHCO_3$, water and brine. The organic layer was dried over Na_2SO_4, the solvent removed under reduced pressure and the product purified by column chromatography on silica.

General Procedure 4; Deacetylation with sodium methanolate

The peracetylated 2-(benzyloxycarbonyl)aminoethyl glucoside was dissolved in methanol and NaOMe in methanol (0.33 equiv) added. The reaction was then stirred for 3 h to o/n at r.t. The base was neutralised with Amberlite IR-120, the resin removed by filtration and the solvent evaporated *in vacuo* to yield the product.

General Procedure 5; Hydrogenolysis of *N*-Cbz-protecting groups

The 2-(benzyloxycarbonyl)aminoethyl glycoside was dissolved in MeOH and Pd/C (10 %) added. The reaction was then stirred under a H_2 atmosphere for 2 h to o/n. The solution was then filtered through Celite and the solvent removed under reduced pressure to yield the free amine.

Synthesis of aminoethyl xyloside 1

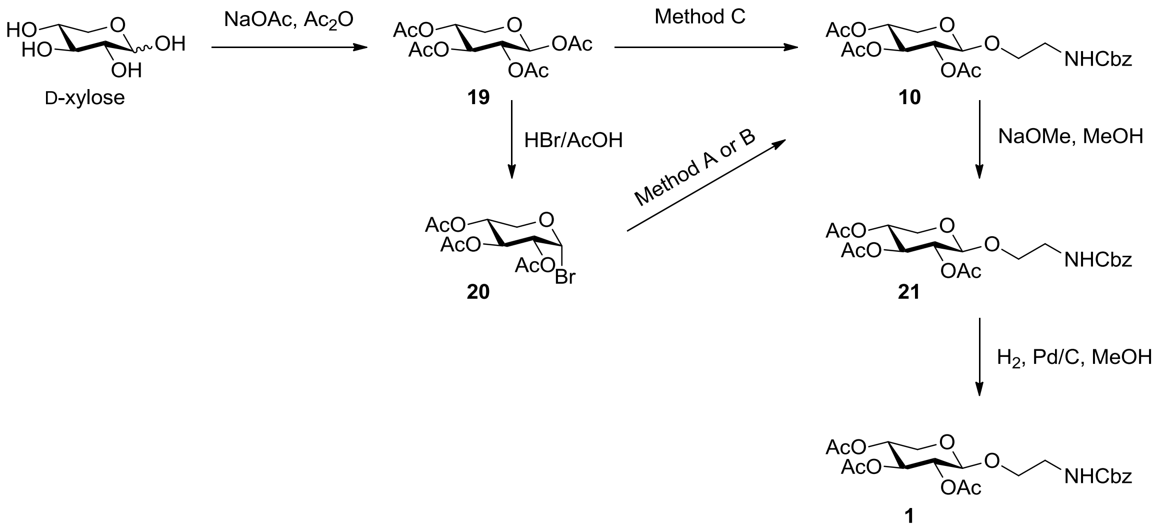

1,2,3,4-Tetra-*O*-acetyl-β-D-xylopyranose (19)

To a solution of D-xylose (5.00g, 33.3 mmol) in Ac_2O (40 mL) was added NaOAc (2.73 g, 33.3 mmol) and the mixture heated at reflux for 4 h. After cooling to r.t., the solution was poured into ice-water (1L) and stirred for 2 h. The solid was collected by filtration, dried and recrystallised from water to yield peracetylated xylose **19** as colourless crystals (8.95 g, 28.1 mmol, 85%).

m.p. = 126–127 °C (water), Lit. [1] 128 °C; $[\alpha]_D^{20}$ = −38.5 (*c* 2.3, CH_2Cl_2); ^{1}H NMR (400 MHz, $CDCl_3$): δ (ppm) = 2.05 (s, 6H, 2 $COCH_3$), 2.05 (s, 3H, $COCH_3$), 2.10 (s, 3H, $COCH_3$), 3.52 (dd, *J* = 8.5, 12.0 Hz, 1H, 5-H_a), 4.14 (dd, *J* = 5.0, 12.0 Hz, 1H, 5-H_e), 4.97 (dt, *J* = 5.0, 8.3 Hz, 1H, 4-H), 5.03 (dd, *J* = 6.9, 8.4 Hz, 1H, 2-H), 5.20 (t, *J* = 8.3 Hz, 1H, 3-H), 5.71 (d, *J* = 6.9 Hz, 1H, 1-H); ^{13}C NMR (101 MHz, $CDCl_3$) δ (ppm) = 20.75, 20.81, 20.9, 21.0 (4 q, 4 $COCH_3$), 62.9 (t, C-5), 68.4 (d, C-4), 69.6 (d, C-2), 71.1 (d, C-3), 92.2 (d, C-1), 169.2, 169.5, 170.0 (3 s, 4 $COCH_3$); IR: $\tilde{\nu}$ (cm^{-1}) = 1753, 1433, 1366, 1217, 1083, 1042, 965, 912, 882, 600; HRMS (ESI+): *m/z* calcd for $C_{13}H_{18}O_9$ $[M+Na]^+$ 341.0849, found 341.0854.

2,3,4-Tri-*O*-acetyl-α-D-xylopyranosyl bromide (20)

Peracetylated xylose **19** (1.00 g, 3.14 mmol) was dissolved in abs. CH_2Cl_2 (7 mL) and 0.2 mL Ac_2O added. The solution was cooled to 0 °C and HBr in acetic acid (2 mL, 33 % solution) added dropwise. After stirring for 1 h at 0 °C and 2 h at r.t., the solution was diluted with CH_2Cl_2 (25 mL), washed successively with water (10 mL), sodium bicarbonate (2 x 10 mL)

and brine (10 mL), dried over $MgSO_4$ and concentrated to dryness. The crude product (0.99 g, 93 %) was recrystallised from EtOAc/hexane to give the bromide **20** as colourless crystals (0.886 g, 2.61 mmol, 84 %).

m.p. = 97–99 °C, Lit. [2] m.p. = 101–102 °C ($^{i}Pr_2O$); ^{1}H NMR (400 MHz, $CDCl_3$): δ (ppm) = 2.06 (s, 3H, $COCH_3$), 2.06 (s, 3H, $COCH_3$), 2.11 (s, 3H, $COCH_3$), 3.88 (t, *J* = 11.2 Hz, 1H, 5-H_a), 4.06 (dd, *J* = 6.0, 11.3 Hz, 1H, 5-H_e), 4.78 (dd, *J* = 4.0, 10.0 Hz, 1H, 2-H), 5.04 (ddd, *J* = 6.0, 9.6, 10.9 Hz, 1H, 4-H), 5.57 (t, *J* = 9.8 Hz, 1H, 3-H), 6.59 (d, *J* = 4.0 Hz, 1H, 1-H); ^{13}C NMR (101 MHz, $CDCl_3$) δ (ppm) = 20.66, 20.68, 20.70 (3 q, 3 $COCH_3$), 62.5 (t, C-5), 68.1 (d, C-4), 69.5 (d, C-3), 70.9 (d, C-2), 87.6 (d, C-1), 169.8, 169.9 (2 s, 3 $COCH_3$)

2-(Benzyloxycarbonyl)aminoethyl 2,3,4-tri-*O*-acetyl-β-D-xylopyranoside (10)

Method A:

Following the General Procedure 1, the xylopyranoside **10** was prepared in 2 h using 678 mg (2.00 mmol) bromide **9**, 781 mg (4.00 mmol) *N*-Cbz-aminoethanol and 556 mg (2.20 mmol) $Hg(CN)_2$ in 8 mL acetonitrile. Chromatography (EtOAc/hexane 40:60) yield 208 mg (0.467 mmol, 23 %) of a mixture of both anomers and 612 mg (1.35 mmol, 67 %) of the pure β-anomer **10** (>95:5) as a clear analytically pure oil.

Method B:

From 678 mg (2.00 mmol) bromide **9** in 6 mL acetonitrile according to General Procedure 2; yield after chromatography (EtOAc/hexane 40:60): 168 mg (0.371 mmol, 19 %) of a mixture of both anomers, 526 mg (1.16 mmol, 53 %) of the pure β-anomer **10** (>95:5) as a colourless, spectroscopically pure oil.

Method C:

According to General Procedure 3 starting from 796 mg (2.50 mmol) peracetylated xylose **19** in 15 mL CH_3CN. After an overnight reaction and chromatography (EtOAc/hexane 40:60) 215 mg (0.475 mmol, 19 %) of an anomeric mixture and 589 mg (1.3 mmol, 52 %) of the pure β-anomer **10** were isolated, both as clear oils.

$[\alpha]_D^{20}$ = −61.5 (*c* 3.8, CH_2Cl_2); ^{1}H NMR (400 MHz, $CDCl_3$): δ (ppm) = 2.01 (s, 3H, $COCH_3$), 2.03 (s, 3H, $COCH_3$), 2.05 (s, 3H, $COCH_3$), 3.34 (dd, *J* = 9.1, 11.8 Hz, 1H, 5-H_a), 3.35–3.47 (m, 2H, CH_2NH), 3.63 (m, 1H, $CH_aH_bCH_2NH$), 3.79–3.89 (m, 1H, $CH_aH_bCH_2NH$), 4.09 (dd, *J* = 5.1, 11.7 Hz, 1H, 5-H_e), 4.46 (d, *J* = 7.0 Hz, 1H, 1-H), 4.90 (dd, *J* = 7.0, 8.9 Hz, 1H, 2-H),

4.94 (dt, J = 5.1, 8.9 Hz, 1H, 4-H), 5.10 (s, 2H, CH_2Ph), 5.16 (t, $J_{2,3}$ = 8.8 Hz, 1H, 3-H), 7.28–7.39 (m, 5H, C_6H_5); ^{13}C NMR (101 MHz, $CDCl_3$) δ (ppm) = 20.65, 20.70, 20.74 (3 q, 3 COCH$_3$), 40.8 (t, CH$_2$NH), 62.2 (t, C-6), 66.7 (t, CH$_2$Ph), 68.7 (t, CH$_2$CH$_2$NH), 68.8 (d, C-4), 70.9 (d, C-2), 71.4 (d, C-3), 100.9 (d, C-1), 128.1, 128.2, 128.5 (3 s, *o*-, *m*-, *p*-C from C_6H_5), 136.5 (s, *i*-C from C_6H_5), 156.3 (s, NCOO), 169.6, 169.8, 170.1 (3 s, 3 COCH$_3$); IR: $\tilde{\nu}$ (cm^{-1}) = 3960, 3381, 3069, 2949, 2876, 2646, 2428, 2363, 2255, 2113, 1959, 1753, 1526, 1454, 1428, 1366, 1248, 1222, 1176, 1150, 1078, 1057, 1037, 902, 876; HRMS (ESI+): *m/z* calcd for $C_{21}H_{27}NO_{10}$ $[M+H]^+$ 454.1713, found 454.1728.

2-(Benzyloxycarbonyl)aminoethyl β-D-xylopyranoside (21)

Aminoethyl glycoside **10** (419 mg, 0.924 mmol) was deacetylated as described in General Procedure 4. Yield: 229 mg (0.915 mmol, 99 %), as a white foam.

$[\alpha]_D^{20}$ = −33 (*c* 1.8, MeOH); ^{1}H NMR (400 MHz, $CDCl_3$): δ (ppm) = 3.13 (dd, J = 7.5, 9.1 Hz, 1H, 2-H), 3.14 (dd, J = 10.4, 11.4 Hz, 1H, 5-H$_a$), 3.23 (ddd, J = 4.3, 6.8, 14.0 Hz, 1 H, CH_aH$_b$NH), 3.27 (t, J = 9.0 Hz, 1 H, 3-H), 3.36 (ddd, J = 4.2, 6.2, 14.0 Hz, 1H, CH$_a$$H_b$NH), 3.43 (ddd, J = 5.3, 8.8, 10.4 Hz, 1H, 4-H), 3.54 (ddd, J = 4.2, 7.0, 10.3 Hz, 1H, CH_aH$_b$CH$_2$NH), 3.80 (dd, J = 5.3, 11.4 Hz, 1H, 5-H$_e$), 3.82 (ddd, J = 4.3, 6.2, 10.4 Hz, 1H, CH$_a$$H_bCH_2$NH), 4.16 (d, J = 7.5 Hz, 1H, 1-H), 5.04 (s, 2H, CH_2Ph), 7.21–7.35 (m, 5H, C_6H_5); ^{13}C NMR (101 MHz, $CDCl_3$) δ (ppm) = 41.9 (t, CH$_2$$CH_2$NH), 67.0 (t, C-5), 67.5 (t, CH$_2$Ph), 69.8 (t, CH$_2$CH$_2$NH), 71.2 (d, C-4), 74.9 (d, C-2), 77.7 (d, C-3), 105.2 (d, C-1), 128.8. 129.0, 129.5 (3 d, *o*-, *m*-, *p*-C from C_6H$_5$), 138.3 (s, *i*-C from C_6H_5), 158.9 (s, CO); IR: $\tilde{\nu}$ (cm^{-1}) = 3670–3000, 2939, 2885, 2352, 1698, 1535, 1454, 1421, 1356, 1334, 1263, 1160, 1073, 1046, 981, 894; HRMS (ESI+): *m/z* calcd for $C_{15}H_{21}NO_7$ $[M+Na]^+$ 350.1216, found 350.1217.

2-Aminoethyl β-D-xylopyranoside (1)

From xyloside **21** (520 mg, 1.59 mmol) in MeOH (10 mL) following General Procedure 5. Yield: 303 mg (1.57 mmol, 99 %), as a white foam.

$[\alpha]_D^{20}$ = −36.8 (*c* 2.33, MeOH); ^{1}H NMR (400 MHz, D_2O): δ (ppm) = 2.90–2.91 (m, 2H, CH_2NH$_2$), 3.28 (dd, J = 7.9, 9.2 Hz, 1H, 2-H), 3.33 (dd, J = 10.5, 11.6 Hz, 1H, 5-H$_a$), 3.44 (t, J = 9.2 Hz, 1H, 3-H), 3.62 (ddd, J = 5.5, 9.1, 10.5 Hz, 1H, 4-H), 3.70 (ddd, J = 4.5, 6.4. 10.7 Hz, 1H, CH_aH$_b$CH$_2$NH$_2$), 3.91 (ddd, J = 4.6, 5.8, 10.4 Hz, 1H, CH$_a$$H_bCH_2NH_2$), 3.96 (dd, J = 5.5, 11.6 Hz, 1H, 5-H$_e$), 4.42 (d, J = 7.9 Hz, 1H, 1-H); ^{13}C NMR (101 MHz, D_2O) δ

(ppm) = 43.0 (t, *C*H_2NH_2), 68.0 (t, C-5), 72.1 (d, C-4), 74.2 (t, *C*$H_2CH_2NH_2$), 75.9 (d, C-2), 78.5 (d, C-3), 106.0 (d, C-1); IR: $\tilde{\nu}$ (cm^{-1}) = 3270, 2929, 2876, 1693, 1572, 1498, 1462, 1420, 1362, 1320, 1246, 1162, 1078, 1047, 979, 895; HRMS (ESI+): *m/z* calcd for $C_7H_{15}NO_5$ $[M+Na]^+$ 216.0848, found 216.0841.

Synthesis of aminoethyl glucoside 2

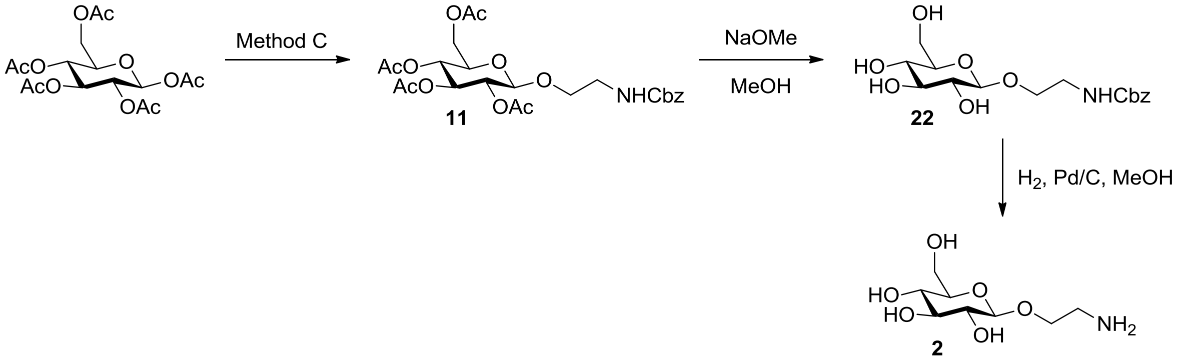

2-(Benzyloxycarbonyl)aminoethyl 2,3,4,6-tetra-*O*-acetyl-β-D-glucopyranoside (11)

Pentaacetyl β-D-glucopyranose (1.00 g, 2.56 mmol) was glycosylated as described in General Procedure Method C. Column chromatography (EtOAc/petroleum ether 40-60 °C 40:60 on silica) gave glucoside **11** as a clear oil (486 mg, 36 %).

$[\alpha]_D^{21}$ = −4.1 (*c* 1.0, $CHCl_3$); ^1H NMR (400 MHz, $CDCl_3$): δ (ppm) = 2.00 (s, 6H, 2 $COCH_3$), 2.03 (s, 3H, $COCH_3$), 2.06 (s, 3H, $COCH_3$), 3.37–3.41 (m, 2H, CH_2NH), 3.68 (ddd, *J* = 2.5, 4.8, 9.9 Hz, 1H, 5-H), 3.69–3.74 (m, 1H, $OCH_aH_bCH_2$), 3.87 (ddd, *J* = 4.1, 5.5, 10.0 Hz, 1H, $OCH_aH_bCH_2$), 4.14 (dd, *J* = 2.4, 12.3 Hz, 6-H_a), 4.14 (dd, *J* = 4.8, 12.4 Hz, 1H, 6-H_b), 4.48 (d, *J* = 8.0 Hz, 1H, 1-H), 4.93 (dd, *J* = 8.0, 9.6 Hz, 1H, 2-H), 5.05 (dd, *J* = 9.4, 9.7 Hz, 1H, 4-H), 5.09 (s, 2H, CH_2Ph), 5.17 (m, 1H, N*H*CBz), 5.19 (dd, *J* = 9.4, 9.6 Hz, 3-H), , 7.33–7.36 (m, 5H, C_6H_5); ^{13}C NMR (101 MHz, $CDCl_3$) δ (ppm) = 20.9, 21.0 (2 q, 4 $COCH_3$), 41.1 (t, CH_2NH), 62.2 (t, C-6), 67.1 (t, CH_2Ph), 68.7 (d, C-4), 69.8 (t, OCH_2CH_2), 71.6 (d, C-2), 72.3 (d, C-5), 73.0 (d, C-3), 101.4 (d, C-1), 128.46, 128.49, 128.9 (3 d, *o*-, *m*-, *p*-C from C_6H_5), 136.8 (s, *i*-C from C_6H_5), 156.7 (s, N*C*OO), 169.7, 169.8, 170.5, 170.9 (4 s, 4 *C*OCH_3); IR: $\tilde{\nu}$ (cm^{-1}) = 3379, 2946, 1755, 1723, 1529, 1431, 1369, 1226, 1039; HRMS (ESI+): *m/z* calcd for $C_{24}H_{31}NO_{12}$ $[M+H]^+$ 526.1925, found 526.1899.

2-(Benzyloxycarbonyl)aminoethyl β-D-glucopyranoside (22)

N-Cbz-aminoethyl-glucoside **11** (370 mg, 0.705 mmol) was deacetylated overnight according to General Procedure 4 to afford **22** as a clear oil (227 mg, 90 %).

$[\alpha]_D^{21}$ = −11.7 (*c* 1.0, MeOD); ^{1}H NMR (400 MHz, MeOD): δ (ppm) = 3.17 (dd, *J* = 7.9, 9.0 Hz, 1H, 2-H), 3.22–3.29 (m, 3H, 4-H, 5-H, CH_aH_bNH), 3.32–3.38 (m, 1H, CH_aH_bNH), 3.34 (t, *J* = 8.7 Hz, 3-H), 3.58 (ddd, *J* = 4.2, 6.9, 10.4 Hz,, 1H, $CH_aH_bCH_2NH$), 3.62 (dd, *J* = 5.2, 12.0 Hz, 1 H, 6-H_b), 3.81 (dd, *J* = 2.0, 11.9 Hz, 1H, 6-H_b), 3.86 (ddd, *J* = 5.6, 7.7, 10.2 Hz, 1H, $CH_aH_bCH_2NH$), 4.22 (d, *J* = 7.8 Hz, 1H, 1-H), 5.02 (s, 2H, CH_2Ph), 7.23–7.29 (m, 5H, C_6H_5); ^{13}C NMR (101 MHz, MeOD) δ (ppm) = 42.7 (t, CH_2NH), 63.3 (t, C-6), 68.2 (t, CH_2Ph), 70.7 (t, CH_2CH_2NH), 72.2 (d, C-5), 75.7 (d, C-2), 78.5 (d, C-3, C-4), 105.1 (d, C-1), 128.2, 128.7, 129.6 (3 d, *o*-, *m*-, *p*-C from C_6H_5), 138.9 (s, *i*-C from C_6H_5), 159.6 (s, NCOO); IR: $\tilde{\nu}$ (cm^{-1}) = 3338, 1702, 1535, 1454, 1337, 1262, 1076, 1031; HRMS (ESI+): *m/z* calcd for $C_{16}H_{23}NO_8$ $[M+Na]^+$ 380.1321, found 380.1318.

2-Aminoethyl β-D-glucopyranoside (2)

2-Benzyloxycarbonylaminoethyl-glucopyranoside **22** (182 mg, 0.51 mmol) was deprotected overnight following the General Procedure 5 for hydrogenation to give aminoethyl glucoside **2** as a white solid (110 mg, 97 %).

$[\alpha]_D^{21}$ = +7.2 (*c* 1.0, MeOH); ^{1}H NMR (400 MHz, MeOD): δ (ppm) = 2.84–2.86 (m, 2H, CH_2NH_2), 3.15 (dd, *J* = 7.8, 9.2 Hz, 1H, 2-H), 3.21–3.24 (m, 2H, 4-H, 5-H), 3.31 (dd, *J* = 9.0, 9.1 Hz, 1H, 3-H), 3.58–3.63 (m, 2H, 6-H_a, $CH_aH_bCH_2NH$), 3.81 (dd, *J* = 1.3, 11.9 Hz, 1H, 6-H_b), 3.88 (ddd, *J* = 5.0, 7.7, 9.9 Hz, 1H, $CH_aH_bCH_2NH$), 4.22 (d, *J* = 7.8 Hz, 1H, 1-H); ^{13}C NMR (101 MHz, MeOD) δ (ppm) = 42.7 (t, CH_2NH_2), 63.5 (t, C-6), 71.5 (t, $CH_2CH_2NH_2$), 72.4 (d, C-5), 75.9 (d, C-2), 78.7 (d, C-3), 78.8 (d, C-4), 105.2 (d, C-1); IR: $\tilde{\nu}$ (cm^{-1}) = 3394, 1645, 1319, 1078, 1039, 614; HRMS (ESI+): *m/z* calcd for $C_8H_{17}NO_6$ $[M+H]^+$ 224.1134, found 224.1135.

Synthesis of aminoethyl galactoside 3

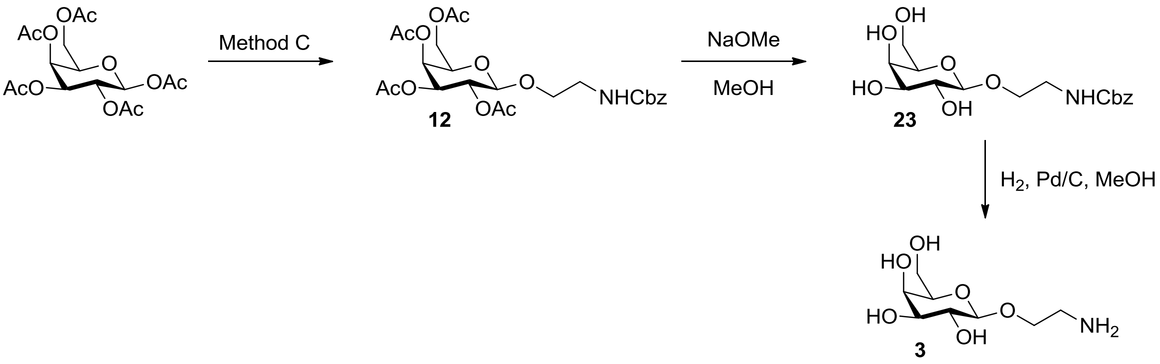

2-(Benzyloxycarbonyl)aminoethyl 2,3,4,6-tetra-*O*-acetyl-β-D-galactopyranoside (12)

Pentaacetyl β-D-galactose (1.95 g, 5.00 mmol) was coupled with *N*-Cbz-ethanolamine in 6 h as described in Method C. The product was purified using column chromatography (ethyl acetate/hexane 50:50) to yield **12** as a clear oil (1.64 g, 62 %).

$[\alpha]_D^{21}$ = −1.4 (*c* 1.0, $CHCl_3$), Lit. [3] $[\alpha]_D^{22}$ = +4.4 (*c* 1.2, CH_2Cl_2), Lit. [4] $[\alpha]_D^{20}$ = +20.7 (*c* 1, CH_2Cl_2); ^{1}H NMR (500 MHz, $CDCl_3$): δ (ppm) = 1.90 (s, 3H, $COCH_3$), 1.93 (s, 3H, $COCH_3$), 1.95 (s, 3H, $COCH_3$), 2.07 (s, 3H, $COCH_3$), 3.32 (m, 2H, CH_2NH), 3.62 (ddd, *J* = 3.6, 7.1, 10.2 Hz, 1H, $CH_aH_bCH_2NH$), 3.80–3.82 (m, 2H, 5-H, $CH_aH_bCH_2NH$), 4.06 (d, *J* = 6.6 Hz, 2H, 6-H_2), 4.38 (d, *J* = 7.9 Hz, 1H, 1-H), 4.93 (dd, *J* = 3.4, 10.5 Hz, 1H, 3-H), 5.02 (s, 2H, CH_2Ph), 5.10 (dd, *J* = 8.0, 10.4 Hz, 1H, 2-H), 5.19 (t, *J* = 5.4 Hz, 1H, N*H*), 5.31 (dd, *J* = 0.7, 3.4 H, 1H, 4-H), 7.22–7.30 (m, 5H, C_6H_5); ^{13}C NMR (126 MHz, $CDCl_3$) δ (ppm) = 20.5–20.7 (4 q, 4 $COCH_3$), 40.8 (t, CH_2NH), 61.3 (t, C-6), 66.7 (t, CH_2Ph), 67.0 (d, C-4), 68.8 (d, C-2), 69.4 (t, OCH_2CH_2NH), 70.7 (d, C-3, C-5), 101.5 (d, C-1), 128.1, 128.5 (2 d, *o*-, *m*-, *p*-C from C_6H_5), 136.5 (s, *i*-C from C_6H_5), 156.3 (s, NCOO), 169.6, 170.1, 170.2, 170.4 (4 s, 4 $COCH_3$); IR: $\tilde{\nu}$ (cm^{-1}) = 3393, 2947, 1743, 1714, 1524, 1371, 1222, 1048; HRMS (ESI+): *m/z* calcd for $C_{24}H_{31}NO_{12}$ $[M+Na]^+$ 548.1744, found 548.1747.

2-(Benzyloxycarbonyl)aminoethyl β-D-galactopyranoside (23)

Tetraacetate **12** (1.62 g, 3.08 mmol) was deacetylated as described above to obtain galactoside **23** in quantitative yield (1.10 g, 3.08 mmol) as a clear oil.

$[\alpha]_D^{21}$ = +1.8 (*c* 1.0, MeOH); ^{1}H NMR (400 MHz, MeOD): δ (ppm) =3.30 (ddd, *J* = 4.2, 6.8, 14.2 Hz, 1H, CH_aH_bNH), 3.40 (ddd, *J* = 4.1, 6.2, 14.2 Hz, 1H, CH_aH_bNH), 3.46 (dd, *J* = 3.2, 9.7 Hz, 1H, 3-H), 3.50 (ddd, *J* = 1.0, 5.3, 6.8 Hz, 1H, 5-H), 3.52 (dd, *J* = 7.3, 9.8 Hz, 1H, 2-

H), 3.63 (ddd, J = 4.0, 6.8, 10.5 Hz, 1H, CH_aH$_b$CH$_2$NH), 3.70 (dd, J = 5.3, 11.4 Hz, 1H, 6-H$_a$), 3.75 (dd, J = 6.9, 11.3 Hz, 1H, 6-H$_b$), 3.82 (dd, J = 1.0, 3.2 Hz, 1H, 4-H), 3.91 (ddd, J = 4.2, 6.2, 10.4 Hz, 1H, CH$_a$$H_bCH_2$NH), 4.22 (d, J = 7.3 Hz, 1H, 1-H), 5.06 (s, 2H, CH_2Ph), 7.24–7.37 (m, 5H, C$_6$$H_5$); ^{13}C NMR (101 MHz, MeOD) δ (ppm) = 42.0 (t, CH$_2$NH), 62.4 (t, C-6), 67.4 (t, CH$_2$Ph), 69.9 (t, CH$_2$CH$_2$NH), 70.2 (d, C-4), 72.5 (d, C-2), 74.8 (d, C-3), 76.6 (d, C-5), 105.0 (d, C-1), 128.8, 129.0, 129.5 (3 d, *o*-, *m*-, *p*-C from C$_6$H$_5$), 138.3 (s, *i*-C from C$_6$H$_5$), 158.9 (s, NCOO); IR: $\tilde{\nu}$ (cm^{-1}) = 3403, 1643, 1264, 1077; HRMS (ESI+): *m/z* calcd for $C_{16}H_{23}NO_8$ [M+Na]$^+$ 380.1316, found 380.1308.

2-Aminoethyl β-D-galactopyranoside (3)

Prepared by hydrogenation (General Procedure 5) from **23** (1.00 g, 3.89 mmol) in MeOH (30 mL) in 6 h. Yield: 806 mg (3.61 mmol, 93 %), as a colourless oil.

$[\alpha]_D^{20}$ = −12.9 (*c* 2.4, MeOH), Lit. [5] $[\alpha]_D$ = −11.3 (*c* 0.23, MeOH); ^{1}H NMR (400 MHz, MeOD): δ (ppm) = 2.80 (ddd, J = 4.2, 6.3, 13.4 Hz, 1H, CH_aH$_b$NH$_2$), 2.84 (ddd, J = 4.4, 5.5, 13.4 Hz, 1H, CH$_a$$H_bNH_2$), 3.45 (dd, J = 3.3, 9.7 Hz, 1H, 3-H), 3.49 (ddd, J = 1.0, 5.3, 7.0 Hz, 1H, 5-H), 3.52 (dd, J = 7.5, 9.7 Hz, 1H, 2-H), 3.61 (ddd, J = 4.4, 6.3, 10.5 Hz, 1H, CH_aH$_b$CH$_2$NH$_2$), 3.69 (dd, J = 5.3, 11.3 Hz, 1H, 6-H$_a$), 3.73 (dd, J = 7.0, 11.3 Hz, 1H, 6-H$_b$), 3.80 (dd, J = 1.0, 3.3 Hz, 1H, 4-H), 3.91 (ddd, J = 4.2, 5.5, 10.3 Hz, 1H, CH$_a$$H_bCH_2NH_2$), 4.21 (d, J = 7.5 Hz, 1H, 1-H); ^{13}C NMR (101 MHz, MeOD) δ (ppm) = 42.2 (t, CH$_2$NH$_2$), 62.5 (t, C-6), 70.3 (d, C-4), 71.9 (t, CH$_2$CH$_2$NH$_2$), 72.6 (d, C-2), 74.9 (d, C-3), 76.7 (d, C-5), 105.1 (d, C-1); IR: $\tilde{\nu}$ (cm^{-1}) = 3320, 3359, 2929, 2887, 1645, 1598, 1073,1042; HRMS (ESI+): *m/z* calcd for $C_8H_{17}NO_6$ [M+H]$^+$ 224.1134, found 224.1133.

Synthesis of aminoethyl mannoside 4

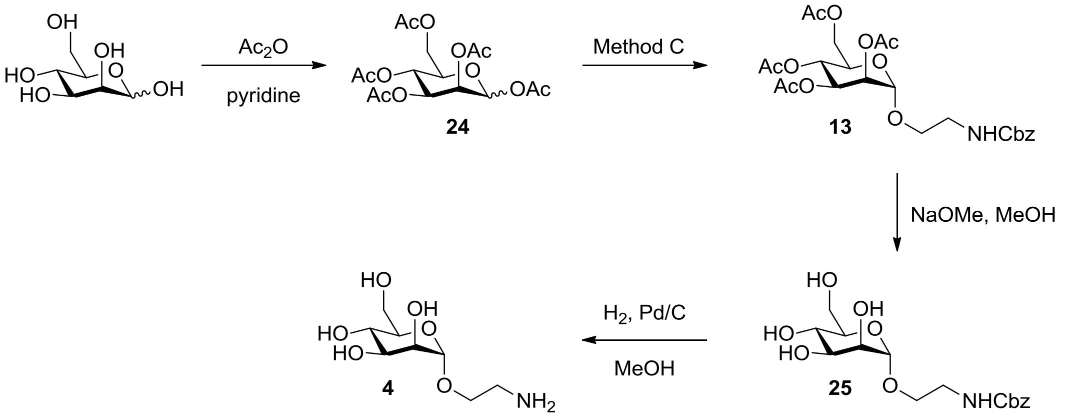

1,2,3,4,6-Penta-*O*-acetyl-D-mannopyranose (24)

D-Mannose (5.00 g, 27.9 mmol) was dissolved in pyridine (35 mL) and acetic anhydride (13 mL) added. After stirring for 16 h at r.t., the reaction mixture was concentrated in vacuum, the residue dissolved in ethyl acetate, washed successively with copper sulphate solution, water and brine, and dried with $MgSO_4$. Ethyl acetate was removed to yield peracetylated mannose **24** (10.6 g, 27.1 mmol, 97 %, mixture of both anomers α/β 33:67) as a clear viscous oil

^{1}H NMR (400 MHz, $CDCl_3$, mixture of both anomers): signals of β-anomer δ (ppm) = 1.98 (s, 3H, $COCH_3$), 2.07 (s, 3H, $COCH_3$), 2.08 (s, 3H, $COCH_3$), 2.15 (s, 3H, $COCH_3$), 2.19 (s, 3H, $COCH_3$), 3.99–4.05 (m, 1H, 5-H), 4.07 (dd, J = 2.4, 12.4 Hz, 1H, 6-H_a), 4.26 (dd, J = 4.9, 12.4 Hz, 1H, 6-H_b), 5.23 (dd, J = 2.0, 3.1 Hz, 1H, 3-H), 5.31–5.34 (m, 2H, 3-H, 4-H), 6.06 (d, J = 2.0 Hz, 1H, 1-H); signals of α-anomer δ (ppm) = 1.98 (s, 3H, $COCH_3$), 2.03 (s, 3H, $COCH_3$), 2.07 (s, 3H, $COCH_3$), 2.15 (s, 3H, $COCH_3$), 2.16 (s, 3H, $COCH_3$), 3.79 (ddd, J = 2.4, 5.3, 9.9 Hz, 1H, 5-H), 4.11 (dd, J = 2.4, 12.4 Hz, 1H, 6-H_a), 4.28 (dd, J = 5.3, 12.4 Hz, 1H, 6-H_b), 5.11 (dd, J = 3.3, 10.0 Hz, 1H, 3-H), 5.27 (t, J = 10.0 Hz, 1H, 4-H), 5.46 (dd, J = 1.2, 3.3 Hz, 1H, 2-H), 5.84 (d, J = 1.2 Hz, 1H, 1-H); ^{13}C NMR (101 MHz, $CDCl_3$, mixture of both anomers): signals of β-anomer: δ (ppm) = 20.73, 20.75, 20.80, 20.86, 20.95 (5 q, 5 $COCH_3$), 62.2 (t, C-6), 65.6 (d, C-4), 68.4 (d, C-2), 68.8 (d, C-3), 70.7 (d, C-5), 90.7 (d, C-1), 168.2, 169.6, 169.8, 170.1, 170.7 (5 s, 5 $COCH_3$); signals of α-anomer: δ (ppm) = 20.63, 20.76, 20.81, 20.84, 20.86 (5 q, 5 $COCH_3$), 62.1 (t, C-6), 65.4 (d, C-4), 68.3 (d, C-2), 70.7 (d, C-3), 73.4 (d, C-5), 90.5 (d, C-1), 168.5, 169.7, 169.9, 170.3, 170.8 (5 s, 5 $COCH_3$).

2-(Benzyloxycarbonyl)aminoethyl 2,3,4,6-tetra-*O*-acetyl-α-D-mannopyranoside (13)

Mannose pentaacetate (**24**, 2.17 g, 5.56 mmol) was glycosylated overnight according to Method C (General Procedure 3) in acetonitrile (25 mL). The product was purified by column chromatography (EtOAc/hexane 40:60 to 50:50) and obtained as a clear oil (1.66 g, 3.17 mmol, 57 %).

$[\alpha]_D^{20}$ = +165 (c 1.8, CH_2Cl_2); ^{1}H NMR (400 MHz, $CDCl_3$): δ (ppm) = 2.00 (s, 3H, $COCH_3$), 2.04 (s, 3H, $COCH_3$), 2.09 (s, 3H, $COCH_3$), 2.16 (s, 3H, $COCH_3$), 3.36–3.53 (m, 2H, CH_2NH), 3.58 (ddd, J = 3.6, 6.8, 10.2 Hz, 1H, $CH_aH_bCH_2NH$), 3.78 (ddd, J = 3.9, 6.2 10.2 Hz, 1H, $CH_aH_bCH_2NH$), 3.97 (ddd, J = 2.3, 5.7 9.5 Hz, 1H, 5-H), 4.08 (dd, J = 2.3, 12.2 H 1H, 6-H_a), 4.26 (dd, J = 5.7, 12.2 Hz, 1H, 6-H_b), 4.82 (d, J = 1.7 Hz, 1H, 1-H), 5.12 (s, 2H, CH_2Ph), 5.20 (bt, J = 5.8 Hz, 1H, NH), 5.25 (dd, J = 1.7, 3.2 Hz, 1H, 2-H), 5.26 (dd, J

= 9.5, 10.1 H), 5.31 (dd, *J* = 3.2, 10.0 Hz, 1H, 3-H), 7.29–7.39 (m, 5H, C_6H_5); ^{13}C NMR (101 MHz, $CDCl_3$) δ (ppm) = 20.7, 20.8, 20.9 (3 q, 4 COccCH_3), 40.7 (CH_2NH), 62.5 (t, C-6), 66.1 (d, C-4), 66.9 (t, CH_2Ph), 67.8 (t, CH_2CH_2NH), 68.8 (d, C-5), 69.0 (d, C-3), 69.4 (d, C-2), 97.8 (d, C-1), 128.2, 128.6 (2 d, *o*-, *m*-, *p*-C from C_6H_5), 136.4 (s, *i*-C from C_6H_5), 156.4 (s, N*C*OO), 169.8, 170.0, 170.1, 170.7 (4 s, 4 *C*OCH_3); IR: $\tilde{\nu}$ (cm^{-1}) = 3391, 2936, 1748, 1531, 1367, 1227, 1140, 1088, 1047, 980; HRMS (ESI+): *m/z* calcd for $C_{24}H_{31}NO_{12}$ $[M+H]^+$ 526.1925, found 526.1913.

2-(Benzyloxycarbonyl)aminoethyl α-D-mannopyranoside (25)

Deprotection of tetraacetate **13** (466 mg, 0.887 mmol) with NaOMe in MeOH (10 mL) according to General Procedure 4 gave 301 mg (0.842 mmol, 95 %) mannoside **25** as a colourless oil.

$[\alpha]_D^{20}$ = +34.7 (*c* 2.3, MeOH); ^{1}H NMR (400 MHz, MeOD): δ (ppm) = 3.27–3.39 (m, 2H, CH_2NH), 3.47–3.55 (m, 2H, 5-H, CH_aH$_b$CH$_2$NH), 3.60 (t, *J* = 9.5 Hz, 1H, 4-H), 3.68 (dd, *J* = 5.8, 11.7 Hz, 1H, 6-H$_a$), 3.69 (dd, *J* = 3.4, 9.3 Hz, 1H, 3-H), 3.74 (ddd, *J* = 4.9, 6.4, 10.2 Hz, 1H, CH$_a$$H_bCH_2$NH), 3.80 (dd, *J* = 1.7, 3.4 Hz, 1H, 2-H), 3.81 (dd, *J* = 2.3, 11.7 Hz, 1H, 6-H$_b$), 4.75 (d, *J* = 1.6 Hz, 1H, 1-H), 5.06 (s, 2H, CH_2Ph) , 7.24–7.36 (m, 5H, C$_6$$H_5$); ^{13}C NMR (101 MHz, MeOD) δ (ppm) = 41.7 (t, CH$_2$*C*H$_2$NH), 62.8 (t, C-6), 67.5 (t, *C*H$_2$Ph, *C*H$_2$CH$_2$NH), 68.5 (d, C-4), 72.0 (d, C-2), 72.5 (d, C-3), 74.7 (d, C-5), 101.6 (d, C-1), 128.8, 129.0, 129.5 (3 s, *o*-, *m*-, *p*-C from C_6H_5), 138.3 (s, *i*-C from C_6H_5), 158.9 (s, N*C*OO); IR: $\tilde{\nu}$ (cm^{-1}) = 3300, 2929, 1698, 1535, 1451, 1409, 1335, 1262, 1136, 1094, 1060, 1027, 975, 912, 880; HRMS (ESI+): *m/z* calcd for $C_{16}H_{23}NO_8$ $[M+Na]^+$ 380.1321, found 380.1316.

2-Aminoethyl α-D-mannopyranoside (4)

Prepared from *N*-Cbz derivative **25** (285 mg, 0.798 mmol) by hydrogenation in MeOH (8 mL) as described above in 93 % yield (166 mg, 0.744 mmol).

$[\alpha]_D^{20}$ = +66 (*c* 2.7, MeOD); ^{1}H NMR (400 MHz, MeOD): δ (ppm) = 2.82–2.86 (m, 2H, CH_2NH$_2$), 3.48 (ddd, *J* = 4.7, 5.9, 10.2 Hz, 1H, CH_aH$_b$CH$_2$NH$_2$), 3.56 (ddd, *J* = 2.1, 5.8, 9.7 Hz, 1H, 5-H), 3.63 (t, *J* = 9.4 Hz, 1H, 4-H), 3.73 (dd, *J* = 5.8, 11.8 Hz, 1H, 6-H$_a$), 3.74 (dd, *J* = 3.4, 9.1 Hz, 1H, 3-H), 3.79 (ddd, *J* = 4.7, 5.9, 10.2 Hz, 1H, CH$_a$$H_bCH_2NH_2$), 3.86 (dd, *J* = 1.7, 3.4 Hz, 1H, 2-H), 3.86 (dd, *J* = 2.1, 11.8 Hz, 1H, 6-H$_b$), 4.80 (d, *J* = 1.7 Hz, 1H, 1-H); ^{13}C NMR (101 MHz, MeOD) δ (ppm) = 101.7 (d, C-1), 74.6 (d, C-5), 72.5 (d, C-4), 72.0 (d, C-2), 70.0 (t, *C*H$_2$CH$_2$NH$_2$), 68.6 (d, C-3), 62.8 (t, C-6), 42.0 (t, *C*H$_2$NH$_2$); IR: $\tilde{\nu}$

(cm^{-1}) = 3350, 2928, 1645, 1598, 1454, 1418, 1361, 1320, 1258, 1207, 1134, 1062, 975, 877, 805; HRMS (ESI+): *m/z* calcd for $C_8H_{17}NO_6$ $[M+H]^+$ 224.1134, found 224.1144.

Synthesis of aminoethyl fucoside 5

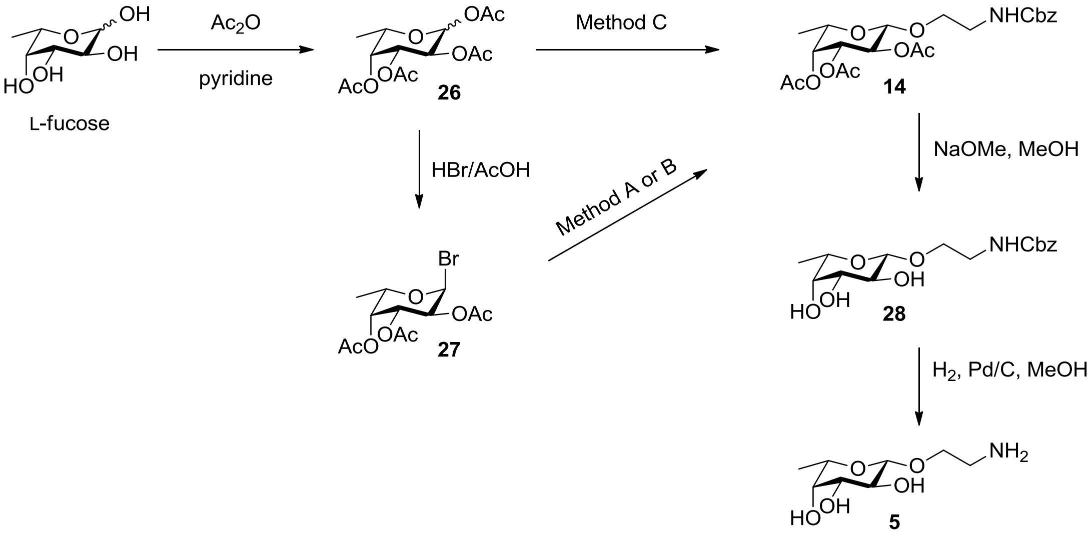

1,2,3,4-Tetra-*O*-acetyl-L-fucopyranose (26)

A solution of L-fucose (1.64 g, 10.0 mmol) in pyridine (18 mL) was cooled to 0 °C and Ac_2O (12 mL) added. The reaction mixture was stirred at 0 °C overnight and then evaporated to dryness. The residue was dissolved in CH_2Cl_2 (40 mL), washed successively with $NaHCO_3$ solution, $CuSO_4$ solution, water and brine, dried over $MgSO_4$ and evaporated. The peracetylated fucose **26** (3.21 g, 9.66 mmol, 97%, mixture of anomers α:β 88:12) was obtained as a colourless oil.

^{1}H NMR (400 MHz, $CDCl_3$, mixture of anomers α/β 88:12), signal of α-anomer: δ (ppm) = 1.09 (d, *J* = 6.5 Hz, 3H, 6-H), 1.94 (s, 3H, COC*H*$_3$), 1.95 (s, 3H, COC*H*$_3$), 2.08 (s, 3H, COC*H*$_3$), 2.12 (s, 3H, COC*H*$_3$), 4.21 (q, *J* = 6.5 Hz, 1H, 5-H), 5.23–5.29 (m, 3 H, 2-H, 3-H, 4-H), 6.27 (d, *J* = 2.9 Hz, 1H, 1-H); ^{13}C NMR (101 MHz, $CDCl_3$, mixture of anomers α/β 88:12), signal of α-anomer: δ (ppm) = 15.9 (q, C-6), 20.60, 20.64, 20.7, 21.0 (4 q, 4 CO*C*H$_3$), 66.5 (d, C-2 or C-3), 67.3 (d, C-5), 67.8 (d, C-2 or C-3), 70.6 (d, C-4), 90.0 (d, C-1), 169.2, 170.0, 170.2, 170.6 (4 s, 4 *C*OCH$_3$); IR: $\tilde{\nu}$ (cm^{-1}) = ; HRMS (ESI+): *m/z* calcd for $C_{14}H_{20}O_9$ $[M+Na]^+$ 355.1005, found 355.1009.

2,3,4-Tri-*O*-acetyl-α-L-fucopyranosyl bromide (27)

L-Fucose tetraacetate **26** (1.68 g, 5.06 mmol) was dissolved in abs. CH_2Cl_2 (12 mL) and acetic anhydride (0.2 mL) added. The solution was cooled to 0 °C, HBr solution in acetic acid (2 mL, 33%) added dropwise and the mixture stirred for 0.5 h at 0 °C and 2 h at r.t. The solution was diluted with CH_2Cl_2 (20 mL), washed successively with water, $NaHCO_3$ solution (2 x) and brine, dried over $MgSO_4$ and concentrated *in vacuo*. The bromide **27** was isolated as a yellowish oil (1.55 g, 4.39 mmol, 87 %) and used without further purification.

^{1}H NMR (400 MHz, $CDCl_3$): δ (ppm) = 1.22 (d, *J* = 6.5 Hz, 3H, 6-H), 2.02 (s, 3H, COCH_3), 2.11 (s, 3H, COCH_3), 2.18 (s, 3H, COCH_3), 4.41 (ddq, *J* = 0.8, 1.3, 6.5 Hz, 1H, 5-H), 5.03 (dd, *J* = 3.9, 10.6 Hz, 1H, 2-H), 5.36 (dd, *J* = 1.3, 3.3 Hz, 1H, 4-H), 5.41 (dd, *J* = 3.3, 10.6 Hz, 1H, 3-H), 6.70 (d, *J* = 3.9 Hz, 1H, 1-H); ^{13}C NMR (101 MHz, $CDCl_3$) δ (ppm) = 15.7 (q, C-6), 20.7, 20.8, 20.9 (3 q, 3 COCH_3), 68.0 (d, C-2), 68.5 (d, C-3), 70.0, 70.1 (2 d, C-4, C-5), 89.4 (d, C-1), 170.0, 170.3, 170.4 (3 s, C *C*OCH_4).

2-(Benzyloxycarbonyl)aminoethyl 2,3,4-tri-*O*-acetyl-β-L-fucopyranoside (14)

Method A:

Following the General Procedure 1 fucosyl bromide **27** (805 mg, 2.28 mol) was glycosylated in acetonitrile (12 mL) for 2 h at 60 °C. Flash column chromatography (EtOAc/hexane 40:60 to 50:50) of the crude product yield 202 mg (0.432 mmol, 19 %) of a mixture of both anomers and 799 mg (1.71 mmol, 75 %) of the pure β-anomer (>95:5) as a clear oil.

Method B:

Bromide **27** (680 mg, 1.93 mmol) was coupled with *N*-Cbz-aminoethanol in CH_3CN (4 mL) according to General Procedure 2. Column chromatography (EtOAc/hexane 40:60 to 50:50) gave 180 mg (0.385 mmol, 20 %) of a mixture of both anomers and 677 mg (1.45 mmol, 75 %) of the pure β-fucoside **14** (>95:5) as a clear oil.

$[\alpha]_D^{20}$ = −11.6 (*c* 2.4, CH_2Cl_2); ^{1}H NMR (400 MHz, $CDCl_3$): δ (ppm) = 1.21 (d, *J* = 6.4 Hz, 1H, 6-H), 1.98 (s, 3H, COCH_3), 1.99 (s, 3H, COCH_3), 2.17 (s, 3H, COCH_3), 3.30–3.48 (m, 2H, CH_2NH), 3.67 (ddd, *J* = 3.6, 7.4, 9.8 Hz, 1H, C$H_a$$H_b$$CH_2$NH), 3.79 (dq, *J* = 0.9, 6.4 Hz, 1H, 5-H), 3.89 (ddd, *J* = 3.8, 5.8, 9.8 Hz, 1H, $CH_a$$H_b$$CH_2$NH), 4.42 (d, *J* = 7.9 Hz, 1H, 1-H), 5.00 (dd, *J* = 3.5, 10.5 Hz, 1H, 3-H), 5.10 (s, 2H, CH_2Ph), 5.16 (dd, *J* = 7.9, 10.5 Hz, 1H, 2-H), 5.23 (dd, *J* = 0.9, 3.5 Hz, 1H, 4-H), 7.29–7.37 (m, 5 H, C_6H_5); ^{13}C NMR (101 MHz,

$CDCl_3$) δ (ppm) = 16.0 (q, C-6), 20.72, 20.70, 20.6 (3 q, 3 CO*C*H_3), 40.8 (t, *C*H_2NH), 66.7 (t, *C*H_2Ph), 68.9 (d, C-2), 69.3 (d, C-5), 69.4 (t, *C*H_2CH_2NH), 70.1 (d, C-4), 71.2 (d, C-3), 101.4 (d, C-1), 128.11, 128.14, 128.5 (3 d, *o*-, *m*-, *p*-C from C_6H_5), 136.5 (s, *i*-C from C_6H_5), 156.3 (s, N*C*OO), 169.7, 170.2, 170.7 (3 s, 3 *C*OCH_3); IR: $\tilde{\nu}$ (cm^{-1}) = 3380, 2990, 2938, 2876, 1748, 1526, 1454, 1433, 1370, 1253, 1223, 1176, 1135, 1068, 934, 908; HRMS (ESI+): *m/z* calcd for $C_{22}H_{29}NO_{10}$ $[M+H]^+$ 468.1870, found 468.1882.

2-(Benzyloxycarbonyl)aminoethyl β-L-fucopyranoside (28)

Peracetylated fucoside **14** (633 mg, 1.35 mmol) was deprotected in MeOH (10 mL) as described in General Procedure 4. The product **28** was obtained as a colourless viscous oil (454 mg, 1.33 mmol, 99 %).

$[\alpha]_D^{20}$ = −3.9 (*c* 3.27, MeOH); ^1H NMR (400 MHz, MeOD): δ (ppm) = 1.24 (d, *J* = 6.5 Hz, 3H, 6-H), 3.24–3.31 (m, 1H, CH_aH_bNH), 3.38 (ddd, *J* = 4.0, 6.3, 13.9 Hz, 1H, CH_aH_bNH), 3.44–3.49 (m, 2H, 2-H, 3-H), 3.56–3.62 (m, 3H, 4-H, 5-H, C$H_aH_bCH_2$NH), 3.85 (ddd, *J* = 4.1, 6.43, 10.5 Hz, 1H, C$H_aH_bCH_2$NH), 4.18 (d, *J* = 7.6 Hz, 1H, 1-H), 5.06 (s, 2H, *C*H_2Ph), 7.25–7.36 (m, 5H, C_6H_5); ^{13}C NMR (101 MHz, $CDCl_3$) δ (ppm) = 16.7 (q, C-6), 42.0 (t, *C*H_2NH), 67.5 (t, *C*H_2Ph), 69.9 (t, *C*H_2CH_2NH), 71.9, 72.3, 72.9, 74.9 (4 d, C-2, C-3, C-4, C-5), 104.9 (d, C-1), 128.9, 129.0, 129.5 (3 d, *o*-, *m*-, *p*-C from C_6H_5), 138.3 (s, *i*-C from C_6H_5), 158.9 (s, CO); IR: $\tilde{\nu}$ (cm^{-1}) = 3667–3010, 2982, 2939, 2885, 2352, 1698, 1535, 1454, 1416, 1377, 1334, 1258, 1171, 1133, 1073, 997, 948, 900, 845; HRMS (ESI+): *m/z* calcd for $C_{16}H_{23}NO_7$ $[M+H]^+$ 342.1553, found 342.1550.

2-(Benzyloxycarbonyl)aminoethyl β-L-fucopyranoside (5)

N-Cbz-deprotection of **28** (421 mg, 1.23 mmol) according to General Procedure 5 afford free fucoside **5** (244 mg, 1.18 mmol, 96 %) as a yellowish foam.

$[\alpha]_D^{20}$ = −17.1 (*c* 0.93, MeOH); ^1H NMR (400 MHz, D_2O): δ (ppm) = 1.26 (d, *J* = 6.5 Hz, 3H, 6-H), 2.81–2.88 (m, 2H, CH_2NH_2), 3.49 (dd, *J* = 7.9, 9.9 Hz, 1H, 2-H), 3.65 (dd, *J* = 3.5, 9.9 Hz, 1H, 3-H), 3.67–3.73 (m, 1H, C$H_aH_bCH_2NH_2$), 3.75 (d, *J* = 3.5 Hz, 1H, 4-H), 3.80 (q, *J* = 6.5 Hz, 1H, 5-H), 3.92 (dt, *J* = 5.4, 10.5 Hz, 1H, C$H_aH_bCH_2NH_2$), 4.39 (d, *J* = 7.9 Hz, 1H, 1-H); ^{13}C NMR (101 MHz, D_2O) δ (ppm) = 18.2 (q, C-6), 43.0 (t, *C*H_2NH_2), 73.4 (d, C-2), 73.8 (d, C-5), 74.17 (t, *C*$H_2CH_2NH_2$), 74.18 (d, C-4), 75.7 (d, C-3), 105.7 (d, C-1); IR: $\tilde{\nu}$ (cm^{-1}) = 3545, 2981, 2939, 2876, 2729, 1645, 1593, 1446, 1378, 1315, 1173, 1136, 1068, 994; HRMS (ESI+): *m/z* calcd for $C_8H_{17}NO_5$ $[M+Na]^+$ 230.1004, found 230.0988.

Synthesis of aminoethyl glucosamine 6

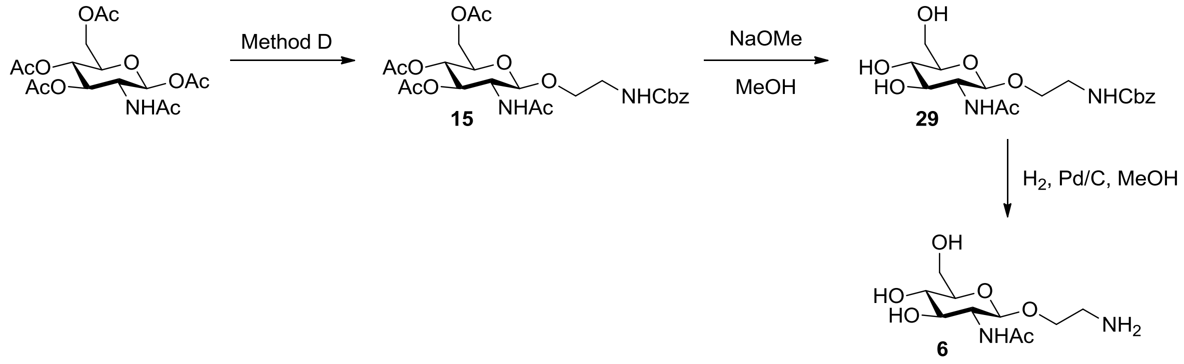

2-(Benzyloxycarbonyl)aminoethyl 2-acetamido-3,4,6-tri-*O*-acetyl-2-deoxy-β-D-gluco-pyranoside (15)

Based on [6], β-D-glucosamine pentaacetate (Acros Organics, 1.00 g, 2.57 mmol) and *N*-Cbz-ethanolamine (1.25 g, 6.43 mmol, 2.5 equiv) were dissolved in dry acetonitrile (10 mL) under a nitrogen atmosphere. The reaction was cooled to 0 °C and $SnCl_4$ (360 µL, 3.08 mmol, 1.2 equiv) added dropwise. The reaction was warmed to room temperature and heated at 75 °C for 14 h. The reaction mixture was allowed to cool to room temperature, quenched with 2 mL Et_3N and concentrated in vacuum. The residue was dissolved in 50 mL dichloromethane, washed with water (20 mL), the organic phase was then dried over $MgSO_4$ and evaporated. Flash column chromatography (EtOAc/hexane 80:20 on silica) gave a white solid which was recrystallised from chloroform/ethyl acetate to yield the product as colourless crystals (820 mg, 1.56 mmol, 61 %).

$[\alpha]_D^{20}$ = −70.1 (*c* 2.1, CH_2Cl_2), Lit. [7] $[\alpha]_D^{24}$ = −15 (*c* 1.0, $CHCl_3$); ^1H NMR (500 MHz, $CDCl_3$): δ (ppm) = 1.82 (s, 3H, $COCH_3$), 1.96 (s, 6H, 2 $COCH_3$), 1.98 (s, 3H, $COCH_3$), 3.21–3.29 (m, 1H, CH_aH_bNH), 3.33–3.42 (m, 1H, CH_aH_bNH), 3.61 (m, 2H, 5-H, $CH_aH_bCH_2NH$), 3.80 (ddd, *J* = 3.5, 5.9, 9.9 Hz, 1H, $CH_aH_bCH_2NH$), 3.84 (dt, *J* = 8.6, 10.2 Hz, 1H, 2-H), 4.06 (dd, *J* = 2.0, 12.3 Hz, 1H, 6-H_a), 4.16 (dd, *J* = 4.8, 12.3 Hz, 1H, 6-H_b), 4.54 (d, *J* = 8.3 Hz, 1H, 1-H), 4.98 (dd, *J* = 9.5, 9.8 Hz, 1H, 4-H), 5.02 (s, 2H, CH_2Ph), 5.12 (dd, *J* = 9.8, 10.2 Hz, 1H, 3-H), 5.31 (t, *J* = 5.2 Hz, 1H, CH_2NH), 5.87 (d, *J* = 8.8 Hz, 1H, 2-N*H*), 7.23–7.31 (m, 5H, C_6H_5); ^{13}C NMR (126 MHz, $CDCl_3$) δ (ppm) = 20.68, 20.73, 20.8, 23.2 (4 q, 4 $COCH_3$), 40.7 (t, CH_2NH), 54.4 (d, C-2), 62.1 (t, C-6), 66.7 (t, CH_2Ph), 68.5 (d, C-4), 69.1 (t, CH_2CH_2NH), 71.8 (d, C-5), 72.4 (d, C-3), 101.1 (d, C-1), 128.1, 128.2, 128.6 (3 d, *o*-, *m*-, *p*-C from C_6H_5), 136.6 (s, *i*-C from C_6H_5), 156.6 (s, NCOO), 169.5, 170.7, 170.9, 171.0 (4 s, 4

COCH$_3$); IR: $\tilde{\nu}$ (cm^{-1}) = 3317, 2949, 1743, 1701, 1660, 1547, 1433, 1377, 1243, 1171, 1150, 1047; HRMS (ESI+): *m/z* calcd for $C_{24}H_{32}N_2O_{11}$ [M+H]$^+$ 525.2084, found 525.2077.

2-(Benzyloxycarbonyl)aminoethyl 2-acetamido-2-deoxy-β-D-glucopyranoside (29)

Prepared from glucosamine **15** (414 mg, 0.789 mmol) following the General Procedure 4 for deacetylation. Yield: 311 mg (0.781 mmol, 99 %), as a white foam.

$[\alpha]_D^{20}$ = −9.6 (*c* 2.1, MeOH), Lit. [7] $[\alpha]_D^{24}$ = −21.5 (*c* 1.0, $CHCl_3$); ^{1}H NMR (400 MHz, MeOD): δ (ppm) = 1.93 (s, 3H, COCH$_3$), 3.23–3.34 (m, 4H, 4-H, 5-H, CH_2NH), 3.43 (dd, *J* = 8.4, 10.3 Hz, 1H, 3-H), 3.58 (ddd, J = 5.4, 5.6, 10.6 Hz, 1H, CH_aH$_b$CH$_2$NH), 3.65 (dd, *J* = 8.4, 10.3 Hz, 1H, 2-H), 3.66 (dd, *J* = 5.6, 11.9 Hz, 1H, 6-H$_a$), 3.84 (m, 1H, CH$_a$$H_bCH_2$NH), 3.86 (dd, *J* = 2.2, 12.0 Hz, 1H, 6-H$_b$), 4.38 (d, *J* = 8.4 Hz, 1H, 1-H), 5.05 (s, 2H, CH_2Ph), 7.35–7.25 (m, 5H, C_6H_5); ^{13}C NMR (101 MHz, MeOD) δ (ppm) = 23.0 (q, COCH$_3$), 41.9 (t, CH$_2$NH), 57.2 (d, C-2), 62.7 (t, C-6), 67.4 (t, CH$_2$Ph), 69.5 (t, CH$_2$CH$_2$NH), 71.9 (d, C-4), 75.9 (d, C-3), 77.9 (d, C-5), 102.8 (d, C-1), 128.9, 129.0, 129.5 (s, *o*-, *m*-, *p*-C from C_6H_5), 138.2 (s, *i*-C from C_6H_5), 158.8 (s, NCOO), 174.0 (s, COCH$_3$); IR: $\tilde{\nu}$ (cm^{-1}) = 3612–3000, 2938, 2886, 1700, 1644, 1546, 1459, 1421, 1372, 1312, 1258, 1149, 1111, 1073, 1035; HRMS (ESI+): *m/z* calcd for $C_{18}H_{26}N_2O_8$ [M+Na]$^+$ 421.1587, found 421.1570.

2-Aminoethyl 2-acetamido-2-deoxy-β-D-glucopyranoside (6)

According to General Procedure 5, *N*-Cbz aminoethyl pyranoside **29** (280 mg, 0.703 mmol) was hydrogenated to yield free GlcNAc **6** (178 mg, 674 mmol, 96 %) as a yellowish foam.

$[\alpha]_D^{20}$ = −28.3 (*c* 1.87, MeOH); ^{1}H NMR (400 MHz, D_2O): δ (ppm) = 2.05 (s, 3H, COCH$_3$), 2.69–2.85 (m, 2H, CH_2NH$_2$), 3.34–3.52 (m, 2H, 4-H, 5-H), 3.52–3.59 (m, 1H, 3-H), 3.59–3.67 (m, 1H, CH_aH$_b$CH$_2$NH$_2$), 3.71–3.80 (m, 2H, 2-H, 6-H$_a$), 3.87–4.01 (m, 2H, 6-H$_b$, CH$_a$$H_bCH_2NH_2$), 4.53 (d, *J* = 8.4 Hz, 1H, 1-H); ^{13}C NMR (101 MHz, D_2O) δ (ppm) = 25.0 (q, COCH$_3$), 43.0 (t, CH$_2$NH$_2$), 58.5 (d, C-2), 63.6 (t, C-6), 72.8 (d, C-5), 74.6 (t, CH$_2$CH$_2$NH$_2$), 76.6 (d, C-3), 78.7 (d, C-4), 104.3 (d, C-1), 177.6 (s, COCH$_3$); IR: $\tilde{\nu}$ (cm^{-1}) = 3320, 3086, 2929, 2876, 2361, 1698, 1651, 1551, 1451, 1430, 1372, 1315, 1262, 1152, 1115, 1073, 1036, 947, 900; HRMS (ESI+): *m/z* calcd for $C_{10}H_{20}N_2O_6$ [M+H]$^+$ 265.1400, found 265.1404.

Synthesis of aminoethyl lactoside 7

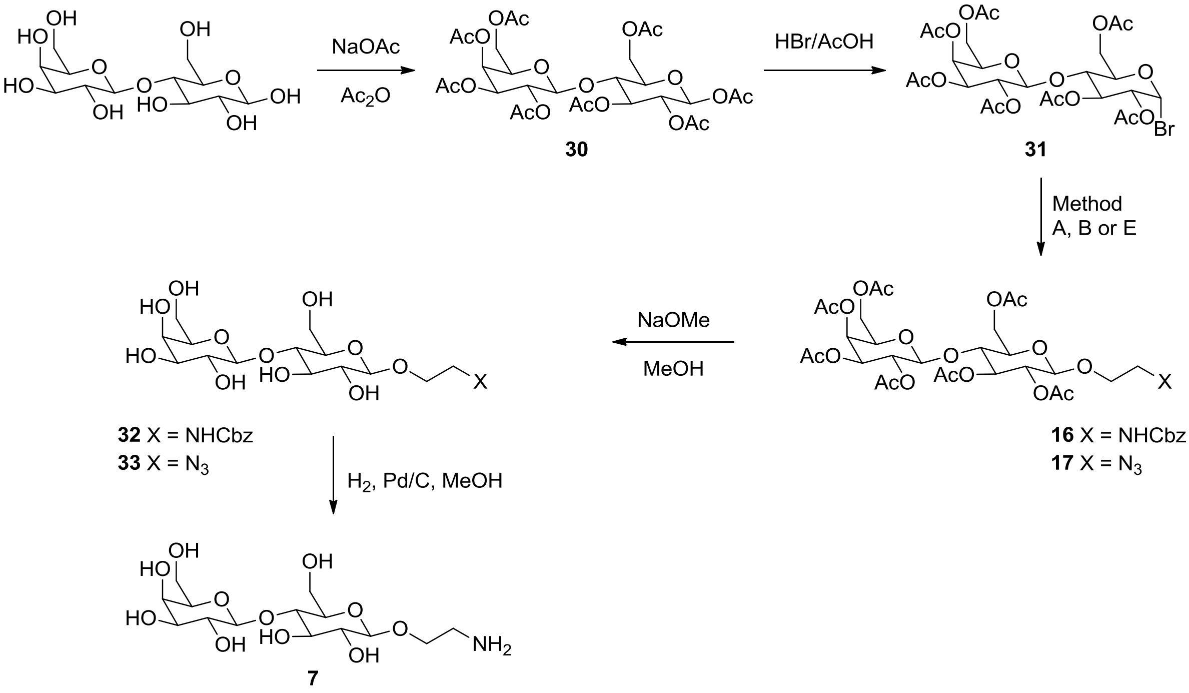

β-D-Lactose octaacetate (30)

D-Lactose (5.00 g, 14.6 mmol) and NaOAc (5.00 g, 61 mmol) were heated in acetic anhydride (90 mL) at reflux. After 4 h, the solution was allowed to cool to r.t. and poured in ice-water (1.5 L). After stirring overnight, the solid was filtered and recrystallised from methanol to yield peracetylated lactose **30** (7.73 g, 11.4 mmol, 78 %) as colourless crystals.

m.p. = 95–96 °C; $[\alpha]_D^{20}$ = +99 (*c* 2.0, CH_2Cl_2); ^{1}H NMR (400 MHz, $CDCl_3$): δ (ppm) = 1.94 (s, 3H, COC*H*$_3$), 2.01 (s, 3H, COC*H*$_3$), 2.02 (s, 3H, COC*H*$_3$), 2.03 (s, 3H, COC*H*$_3$), 2.04 (s, 3H, COC*H*$_3$), 2.07 (s, 3H, COC*H*$_3$), 2.10 (s, 3H, COC*H*$_3$), 2.13 (s, 3H, COC*H*$_3$), 3.74 (ddd, *J* = 2.0, 4.8, 9.9 Hz, 1H, 5-H), 3.82 (dd, *J* = 8.9, 9.9 Hz, 1H, 4-H), 3.85 (ddd, *J* = 1.1, 6.4, 7.2 Hz, 1H, 5'-H), 4.05 (dd, *J* = 7.2, 11.1, Hz, 1H, 6'-H_a), 4.09 (dd, *J* = 4.8, 12.1 Hz, 1 H, 6-H_a), 4.11 (dd, *J* = 6.4, 11.1 Hz, 1 H, 6'-H_b), 4.43 (dd, *J* = 2.0, 12.1 Hz, 1H, 6-H_b), 4.45 (d, *J* = 7.9 Hz, 1H, 1'-H), 4.92 (dd, *J* = 3.5, 10.5 Hz, 1H, 3'-H), 5.02 (dd, *J* = 8.3, 9.5 Hz, 1H, 2-H), 5.08 (dd, *J* = 7.9, 10.5 Hz, 1H, 2'-H), 5.22 (dd, *J* = 8.9, 9.5 Hz, 1H, 3-H), 5.32 (dd, *J* = 1.1, 3.5 Hz, 1H, 4'-H), 5.64 (d, *J* = 8.3 Hz, 1H, 1-H); ^{13}C NMR (101 MHz, $CDCl_3$) δ (ppm) = 20.60, 20.70, 20.73, 20.84, 20.91, 20.93 (6 q, 8 CO*C*H_3), 60.9 (t, C-6'), 61.8 (t, C-6), 66.7 (d, C-4'), 69.0 (d, C-2'), 70.5 (d, C-2), 70.8 (d, C-5'), 71.0 (d, C-3'), 72.7 (d, C-3), 73.5 (d, C-5), 75.7 (d, C-4), 91.6 (d, C-1), 101.0 (d, C-1'), 169.0, 169.2, 169.7, 169.8, 170.2, 170.3, 170.48,

170.51 (8 s, 8 *C*OCH$_3$); IR: $\tilde{\nu}$ (cm^{-1}) = 3550, 2960, 2124, 1753, 1639, 1433, 1372, 1222, 1170, 1073, 1052, 955, 898, 738; HRMS (ESI+): *m/z* calcd for $C_{28}H_{38}O_{19}$ $[M+Na]^+$ 701.1905, found 701.1931.

α-D-Lactosylbromide heptaacetate (31)

Lactose octaacetate **30** (1.50 g, 0.221 mmol) was dissolved in dry chloroform (6 mL) and acetic anhydride (0.2 mL) added. The solution was cooled to 0 °C and HBr in AcOH (2.3 mL, 33 % solution) added. The mixture was stirred for 30 min at 0 °C and 2 h at r.t. The mixture was then diluted with chloroform (20 mL) and washed with sodium bicarbonate (2 × 20 mL) followed by brine (20 mL). The organic layer was dried over $MgSO_4$ and the solvent removed under vacuum. The crude product was recrystallised from chloroform/hexane to yield bromide **31** as colourless needles (1.45 g, 0.207 mmol, 94 %).

m.p. = 143–144 °C ($CHCl_3$/hexane.); $[\alpha]_D^{20}$ = +59 (*c* 1.07, CH_2Cl_2); ^{1}H NMR (400 MHz, $CDCl_3$): δ (ppm) = 1.98 (s, 3H, COC*H*$_3$), 2.06 (s, 3H, COC*H*$_3$), 2.07 (s, 3H, COC*H*$_3$), 2.08 (s, 3H, COC*H*$_3$), 2.10 (s, 3H, COC*H*$_3$), 2.14 (s, 3H, COC*H*$_3$), 2.17 (s, 3H, COC*H*$_3$), 3.82–3.94 (m, 2H, 4-H, 5'H), 4.09 (dd, *J* = 7.2, 11.1 Hz, 1H, 6'-H$_a$), 4.16 (dd, *J* = 5.4, 10.3 Hz, 1H, 6-H$_a$), 4.19–4.23 (m, 2H, 5-H, 6'-H$_b$), 4.47–4.54 (m, 1H, 6-H$_a$), 4.49–4.53 (m, 1H, 6-H$_b$), 4.51 (d, *J* = 7.9 Hz, 1H, 1'-H), 4.77 (dd, *J* = 4.0, 10.0 Hz, 1H, 2-H), 4.96 (dd, *J* = 3.5, 10.4 Hz, 1H, 3'-H), 5.14 (dd, *J* = 7.9, 10.4 Hz, 1H, 4'-H), 5.36 (dd, *J* = 1.1, 3.5 Hz, 1H, 4'-H), 5.56 (t, *J* = 9.7 Hz, 1H, 3-H), 6.53 (d, *J* = 4.0 Hz, 1H, 1-H); ^{13}C NMR (101 MHz, $CDCl_3$) δ (ppm) = 20.5, 20.69, 20.70, 20.71, 20.82, 20.85 (6 q, 6 CO*C*H$_3$), 60.9 (t, C-6'), 61.0 (t, C-6), 66.6 (d, C-4'), 69.0 (d, C-2'), 69.6 (d, C-3), 70.8 (d, C-5'), 70.9 (d, C-2), 71.0 (d, C-3'), 73.0 (d, C-5), 75.0 (d, C-4), 86.4 (d, C-1), 100.8 (d, C-1'), 169.0, 169.3, 170.0, 170.11, 170.17, 170.20, 170.4 (7 s, 7 *C*OCH$_3$); IR: $\tilde{\nu}$ (cm^{-1}) = 3638, 3561, 3484, 3067, 2968, 2431, 2310, 2123, 1963, 1749, 1645, 1431, 1370, 1326, 1228; HRMS (ESI+): *m/z* calcd for $C_{26}H_{35}BrO_{17}$ $[M+Na]^+$ 721.0955, found 721.0968.

2-(Benzyloxycarbonyl)aminoethyl 2,3,4,6-tetra-*O*-acetyl-β-D-galactopyranosyl-(1→4)-2,3,6-tri-*O*-acetyl-β-D-glucopyranoside (16)

Method A

Bromide **31** (699 mg, 1.00 mmol) was glycosylated in acetonitrile (5 mL) as described in General Procedure 1. The crude reaction mixture was treated with pyridine (3 mL) and acetic anhydride (2 mL). After stirring for 4 h at r.t., the solution was concentrated *in vacuo*. Flash

chromatography on silica (EtOAc/hexane 60:40) gave aminoethyl lactoside **16** (457 mg, 0.562 mmol, 56 %) as a white foam.

Method B

Following the General Procedure 2, lactose bromide **31** (699 mg, 1.00 mmol) was glycosylated in acetonitrile (2.5 mL). The crude reaction mixture was dissolved in pyridine (3 mL) and acetic anhydride (2 mL) added. After stirring for 4 h at r.t., the mixture vas concentrated in vacuum and the product isolated and purified by column chromatography (EtOAc/hexane 60:40). Product **16** (699 mg, 0.859 mmol, 86 %) was obtained as a white foam.

Method E

Mercury(II) bromide (180 mg, 0.500 mmol, 0.5 equiv), mercury(II) cyanide (126 mg, 0.500 mmol, 0.5 equiv) and *N*-Cbz-ethanolamine (390 mg, 2.00 mmol, 2 equiv) were dissolved in dry acetonitrile (6 mL) under a nitrogen atmosphere. Lactosyl bromide **31** (700 mg, 1.00 mmol) dissolved in dry acetonitrile (3 mL) was added dropwise to the stirring Hg-salts solution. The reaction mixture was stirred for 16 h at r.t., then diluted with CH_2Cl_2 (50 mL) and washed successively with water (20 mL), $NaHCO_3$ solution (20 mL) and brine (20 mL). The organic layer was dried with $MgSO_4$ and the solvent removed under vacuum. The crude mixture was acetylated using 3:1 pyridine/acetic anhydride (10 mL) overnight. After concentration *in vacuo*, the product was isolated by flash column chromatography (EtOAc/hexane 60:40) to yield lactoside **16** as a clear oil (480 mg, 0.59 mmol, 59 %).

$[\alpha]_D^{20}$ = +77.3 (*c* 1.73, CH_2Cl_2); ^{1}H NMR (400 MHz, $CDCl_3$): δ (ppm) = 1.97 (s, 3H, $COCH_3$), 2.00 (s, 3H, $COCH_3$), 2.04 (s, 3H, $COCH_3$), 2.04 (s, 3H, $COCH_3$), 2.06 (s, 3H, $COCH_3$), 2.08 (s, 3H, $COCH_3$), 2.15 (s, 3H, $COCH_3$), 3.38 (m, 2H, CH_2NH), 3.58 (ddd, *J* = 2.1, 5.2, 9.8 Hz, 1H, 5-H), 3.64–3.74 (m, 1H, $CH_aH_bCH_2NH$), 3.78 (dd, *J* = 9.1, 9.8 Hz, 1H, 4-H), 3.79–3.84 (m, 1H, $CH_aH_bCH_2NH$), 3.87 (ddd, *J* = 1.1, 6.4, 7.4 Hz, 1H, 5'-H), 4.06 (dd, *J* = 5.2, 11.9 Hz, 1H, 6-H_a), 4.07 (dd, *J* = 7.4, 11.2 Hz, 1H, 6'-H_a), 4.13 (dd, *J* = 6.4, 11.2 Hz, 1H, 6'-H_b), 4.44 (d, *J* = 8.0 Hz, 1H, 1-H), 4.47 (d, *J* = 7.8 Hz, 1H, 1'-H), 4.50 (dd, *J* = 2.1, 11.9 Hz, 1H, 6-H_b), 4.87 (dd, *J* = 8.0, 9.6 Hz, 1H, 2-H), 4.95 (dd, *J* = 3.5, 10.4 Hz, 1H, 3'-H), 5.09 (s, 2H, CH_2Ph), 5.11 (dd, *J* = 7.8, 10.4 Hz, 1H, 2'-H), 5.18 (dd, *J* = 9.1, 9.6 Hz, 1H, 3-H), 5.35 (dd, *J* = 1.1, 3.5 Hz, 1H, 4'-H), 7.29–7.37 (m, 5H, C_6H_5); ^{13}C NMR (101 MHz,

$CDCl_3$) δ (ppm) = 20.5 20.7, 20.8, 21.1 (4 q, 7 CO*C*H_3), 40.9 (t, *C*H_2NH), 60.8 (t, C-6'), 61.9 (t, C-6), 66.6 (d, C-4'), 66.7 (t, *C*H_2Ph), 69.1 (d, C-2'), 69.6 (t, *C*H_2CH_2NH), 70.7 (d, C-5'), 71.0 (d, C-3'), 71.6 (d, C-2), 72.6, 72.7 (2 d, C-3, C-5), 76.2 (d, C-4), 100.9, 101.1 (2 d, C-1, C-1'), 128.15, 128.18, 128.5 (3 d, *o*-, *m*-, *p*-C from C_6H_5), 136.5 (s, *i*-C from C_6H_5), 156.3 (s, NCOO), 169.1, 169.7, 169.8, 170.1, 170.2, 170.36, 170.38 (7 s, 7 *C*OCH_3); IR: $\tilde{\nu}$ (cm^{-1}) = 3381, 2949, 1748, 1526, 1430, 1370, 1227, 1170, 1135, 1057, 954, 903; HRMS (ESI+): *m/z* calcd for $C_{36}H_{47}NO_{20}$ $[M+Na]^+$ 836.2589, found 836.2584.

2-Azidoethyl 2,3,4,6-tetra-*O*-acetyl-β-D-galactopyranosyl-(1→4)-2,3,6-tri-*O*-acetyl-β-D-glucopyranoside (17)

To a stirring solution of mercury(II) bromide (202 mg, 0.560 mmol, 0.56 equiv), mercury(II) cyanide (141 mg, 0.560 mmol, 0.56 equiv) and 2-azidoethanol (107 mg, 1.23 mmol, 1.1 equiv) in acetonitrile (5 mL) under a nitrogen atmosphere was added lactose bromide **31** (783 mg, 1.12 mmol) dissolved in 3 mL abs. acetonitrile. After stirring overnight (16 h) at r.t. the solution was diluted with CH_2Cl_2 (50 mL) and washed successively with water (15 mL), $NaHCO_3$ (15 mL), water (15 mL) and brine (15 mL). The organic phase was dried over $MgSO_4$ and concentrated under reduced pressure. Column chromatography on silica (EtOAc/hexane 50:50) gave azide **17** (695 mg, 0.985 mmol, 88 %) as a white solid.

$[\alpha]_D^{20}$ = −196 (*c* 1.33, CH_2Cl_2); ^{1}H NMR (400 MHz, $CDCl_3$): δ (ppm) = 1.97 (s, 3H, COC*H*$_3$), 2.05 (s, 3H, COC*H*$_3$), 2.05 (s, 3H, COC*H*$_3$), 2.05 (s, 3H, COC*H*$_3$), 2.07 (s, 3H, COC*H*$_3$), 2.13 (s, 3H, COC*H*$_3$), 2.16 (s, 3H, COC*H*$_3$), 3.27 (ddd, *J* = 3.3, 5.0, 13.4 Hz, 1H, C$H_aH_bN_3$), 3.48 (ddd, *J* = 3.4, 8.3, 13.4 Hz, 1H, C$H_aH_bN_3$), 3.63 (ddd, *J* = 2.1, 4.9, 9.9 Hz, 1H, 5-H), 3.68 (ddd, *J* = 3.3, 8.3, 10.7 Hz, 1H, C$H_aH_bCH_2N_3$), 3.83 (dd, *J* = 9.1, 9.9 Hz, 1H, 4-H), 3.88 (ddd, *J* = 1.1, 6.4, 7.5 Hz, 1H, 5'-H), 4.00 (ddd, *J* = 3.4, 5.0, 10.7 Hz, 1H, C$H_aH_bCH_2N_3$), 4.05–4.17 (m, 3H, 6-H_a, 6'-H_a, 6'-H_b), 4.50 (d, *J* = 7.9 Hz, 1H, 1'-H), 4.53 (dd, *J* = 2.1, 12.1 Hz, 1H, 6-H_b), 4.56 (d, *J* = 7.9 Hz, 1H, 1-H), 4.93 (dd, *J* = 7.9, 9.4 Hz, 1H, 2-H), 4.96 (dd, *J* = 3.4, 10.5 Hz, 1H, 3'-H), 5.12 (dd, *J* = 7.9, 10.5 Hz, 1H, 2'-H), 5.22 (dd, *J* = 9.1, 9.4 Hz, 1H, 3-H), 5.35 (dd, *J* = 1.1, 3.5 Hz, 1H, 4'-H); ^{13}C NMR (101 MHz, $CDCl_3$) δ (ppm) = 20.53, 20.65, 20.67, 20.74, 20.81, 20.88 (6 q, 6 CO*C*H_3), 50.5 (t, CH_2*C*H_2N_3), 60.8 (t, C-6'), 61.8 (t, C-6), 66.6 (d, C-4'), 68.7 (t, *C*$H_2CH_2N_3$), 69.1 (d, C-2'), 70.7 (d, C-5'), 71.0 (d, C-3'), 71.5 (d, C-2), 72.7 (d, C-5), 72.8 (d, C-3), 76.2 (d, C-4), 100.4 (d, C-1), 101.1 (d, C-1'), 169.1, 169.7, 169.8, 170.1, 170.2, 170.4 (6 s, 7 *C*OCH_3); IR: $\tilde{\nu}$ (cm^{-1}) = 2944, 2880, 2108, 1747, 1646, 1435,

1366, 1235, 1165, 1133, 1050; HRMS (ESI+): *m/z* calcd for $C_{28}H_{39}N_3O_{18}$ $[M+Na]^+$ 728.2126, found 728.2123.

2-(Benzyloxycarbonyl)aminoethyl β-D-galactopyranosyl-(1→4)-β-D-glucopyranoside (32)

Lactoside **16** (650 mg, 0.799 mmo) was deacetylated overnight in MeOH (12 mL) according to General Procedure 4. Yield: 390 mg (0.751 mmol, 94 %), as a white solid.

m.p. = 137–139 °C; $[\alpha]_D^{20}$ = +5.0 (*c* 1.8, MeOH); ^{1}H NMR (400 MHz, MeOD): δ (ppm) = 3.33–3.22 (m, 2H), 3.35–3.43 (m, 2H), 3.47 (dd, *J* = 9.7, 3.3 Hz, 2H), 3.50–3.65 (m, 5H), 3.68 (dd, *J* = 11.4, 4.6 Hz, 1H), 3.73–3.93 (m, 5H), 4.29 (d, *J* = 7.8 Hz, 1H), 4.34 (d, *J* = 7.5 Hz, 1H), 5.06 (s, 2H, CH_2Ph), 7.25–7.37 (m, 5H, C_6H_5); ^{13}C NMR (101 MHz, MeOD) δ (ppm) = 43.0 (t, CH_2NH), 61.8, 62.5 (2 t, C-6, C-6') 67.5 (t, *C*H2PH), 70.0 (t, CH_2CH_2NH), 70.3 (d), 72.5 (d), 74.7 (d), 74.8 (d), 76.2 (d), 76.4 (d), 77.1 (d), 80.4 (d), 105.1, 104.3 (2 d, C-1, C1'), 129.46, 128.98, 128.84 (3 d, *o*-, *m*-, *p*-C from C_6H_5), 138.3 (s, *i*-C from C_6H_5), 158.9 (s, CO); IR: $\tilde{\nu}$ (cm^{-1}) = 3300, 2929, 2876, 2341, 1678, 1540, 1451, 1414, 1372, 1336, 1267, 1157, 1073, 1026, 895; HRMS (ESI+): *m/z* calcd for $C_{22}H_{33}NO_{13}$ $[M+H]^+$ 520.2030, found 520.2014.

2-Azidoethyl β-D-galactopyranosyl-(1→4)-β-D-glucopyranoside (33)

Overnight treatment of azide **17** (326 mg, 0.462 mmol) with NaOMe in MeOH (6 mL) as described in General Procedure 4 gave free lactoside **33** (188 mg, 457 mmol, 99 %) as a white solid.

$[\alpha]_D^{20}$ = +2.9 (*c* 2.73, MeOH); ^{1}H NMR (400 MHz, MeOD): δ (ppm) = 3.30 (dd, *J* = 7.8, 9.0 Hz, 1H, 2-H), 3.45 (ddd, *J* = 2.5, 4.4, 9.3 Hz, 1H, 5-H), 3.47–3.64 (m, 8H, 2-H, 3-H, 4-H, 2'-H, 3'-H, 5'-H, CH_2N_3), 3.72 (dd, *J* = 4.5, 11.4 Hz, 1H, 6'-H_a), 3.74–3.80 (m, 1H, $CH_aH_bCH_2N_3$), 3.80 (dd, *J* = 7.6, 11.5 Hz, 1H, 6'-H_b), 3.84 (dd, *J* = 0.8, 3.2 Hz, 1H, 4'-H), 3.87 (dd, *J* = 4.5, 12.2 Hz, 1H, 6-H_a), 3.94 (dd, *J* = 2.4, 12.1 Hz, 1H. 6-H_b), 4.04 (dt, *J* = 5.0, 10.8 Hz, 1H, $CH_aH_bCH_2N_3$), 4.38 (d, *J* = 7.8 Hz, 1H, 1-H), 4.39 (d, *J* = 7.5 Hz, 2H, 1'-H), 4.93 (s, 2H, CH_2Ph); ^{13}C NMR (101 MHz, MeOD) δ (ppm) = 52.0 (d, CH_2N_3), 61.9 (t, C-6), 62.5 (d, C-6'), 69.4 (t, $CH_2CH_2N_3$), 70.3 (d, C-4'), 72.5 (d, C-2'), 74.7, 74.8 (2 d, C-2, C-3'), 76.3, 76.5, 77.1 (3 d, C-3, C-5, C-5'), 80.6 (d, C-4), 104.3, 105.1 (2 t, C-1, C'-1); IR: $\tilde{\nu}$ (cm^{-1}) = 3300, 2929, 2887, 2110, 1646, 1304, 1157, 1073, 884; HRMS (ESI+): *m/z* calcd for $C_{14}H_{25}N_3O_{11}$ $[M+H]^+$ 412.1567, found 412.1582.

2-Aminoethyl β-D-galactopyranosyl-(1→4)-β-D-glucopyranoside (7)

Aminoethyl lactoside **7** was obtained as a white solid from **16** (260 mg, 0.50 mmol) in MeOH (5 mL) according to General Procedure 5 in 98 % yield (189 mg, 0.49 mmol).

$[\alpha]_D^{20}$ = −1.4 (*c* 2.73, MeOH); ^{1}H NMR (400 MHz, MeOD): δ (ppm) = 2.77–2.85 (m), 3.20–3.30 (m), 3.39 (m), 3.46 (dd, *J* = 3.2, 9.7 Hz), 3.49–3.63 (m), 3.67 (dd, *J* = 4.6, 11.4 Hz), 3.76 (dd, *J* = 7.6, 11.5 Hz), 3.78–3.94 (m), 4.29 (d, *J* = 7.8 Hz), 4.34 (d, *J* = 7.5 Hz); ^{13}C NMR (101 MHz, MeOD) δ (ppm) = 42.2 (t, CH_2NH_2), 61.8 (t), 62.5 (t), 70.3 (d), 72.0 (t), 72.5 (d), 74.76 (d), 74.78 (d), 76.3 (d), 76.5 (d), 77.1 (d), 80.5 (d), 104.4, 105.1 (2 d, C-1, C-1'); IR: $\tilde{\nu}$ (cm^{-1}) = 3555, 2933, 2880, 1694, 1159, 1045, 895; HRMS (ESI+): *m/z* calcd for $C_{14}H_{27}NO_{11}$ $[M+H]^+$ 386.1662, found 386.1649.

Synthesis of aminoethyl maltoside 8

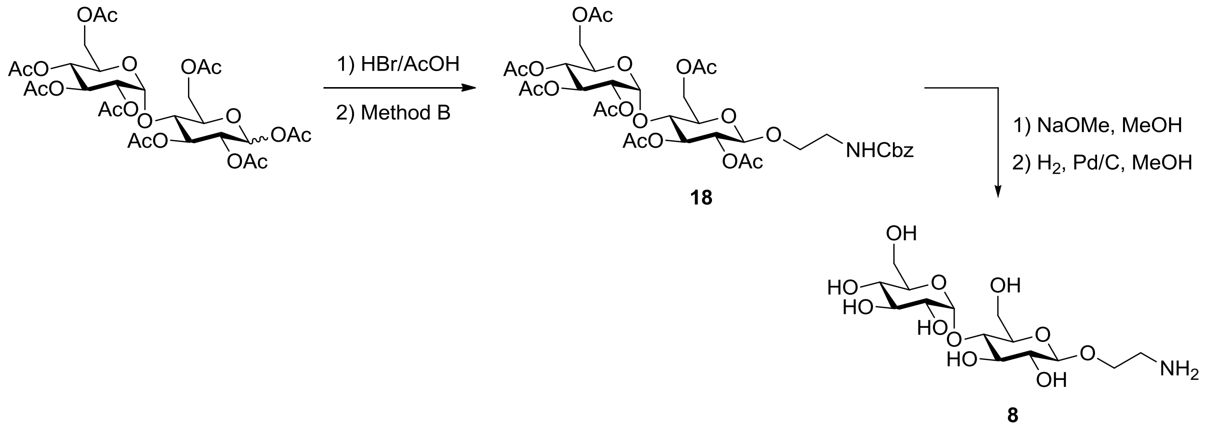

2-(Benzyloxycarbonyl)aminoethyl 2,3,4,6-tetra-*O*-acetyl-α-D-glucopyranosyl-(1→4)-2,3,6-tri-*O*-acetyl-β-D-glucopyranoside (18)

To a solution of β-D-maltose octaacetate (1.00 g, 1.47 mmol) in dry CH_2Cl_2 (5 mL) was added acetic anhydride (0.2 mL) and the solution cooled to 0 °C. HBr in acetic acid (2 mL, 33 %) was added dropwise and the reaction mixture stirred for 0.5 h at 0 °C and 2 h at r.t. The solution was diluted with CH_2Cl_2 (20 mL) and washed successively with water (10 mL), $NaHCO_3$ solution (2x10 mL) and brine (10 mL), dried over $MgSO_4$ and concentrated *in vacuo*. The resulting crude maltose bromide was glycosylated according to General Procedure 2 to yield maltoside **18** (560 mg, 0.69 mmol, 47%) as a white solid.

m.p. = 59–62 °C; $[\alpha]_D^{20}$ = +57.4 (*c* 2.4, $CHCl_3$); ^{1}H NMR (400 MHz, $CDCl_3$): δ (ppm) = 1.91, 1.93, 1.94, 1.96, 1.98, 1.99, 2.03 (6 s, 21H, $COCH_3$), 3.27 - 3.34 (m, 2H, CH_2N), 3.56 (ddd, *J*

= 2.7, 4.2, 9.6 Hz, 1H, 5-H), 3.60 – 3.70 (m, 1H, OCH_aH$_b$), 3.72 – 3.81 (m, 1H, OCH$_a$$H_b$), 3.83 (dd, *J* = 4.2, 10.1 Hz, 1H, 5'-H), 3.9 (t, *J* = 9.1 Hz, 1H, 4-H), 4.0 (dd, *J* = 2.1, 12.4 Hz, 1H, 6'-H$_a$), 4.1 (dd, *J* = 4.4, 12.2 Hz, 1H, 6-H$_a$), 4.2 (dd, *J* = 3.8, 12.5 Hz, 1H, 6'-H$_b$), 4.4 (d, *J* = 8.1 Hz, 2H, 1-H, 6-H$_b$), 4.7 (dd, *J* = 8.1, 9.5 Hz, 1H, 2-H), 4.8 (dd, *J* = 4.0, 10.5 Hz, 1H, 2'-H), 4.99 (t, *J* = 9.9 Hz, 1H, 4'-H), 5.02 – 5.06 (m, 2H, CH_2Ph), 5.17 (t, *J* = 9.2 Hz, 1H, 3-H), 5.28 (t, *J* = 10.0 Hz, 1H, 3'-H), 5.34 (d, *J* = 3.9 Hz, 1H, 1'-H), 7.2 – 7.3 (m, 5H, C$_6$$H_5$); ^{13}C NMR (101 MHz, $CDCl_3$) δ (ppm) = 20.75, 20.77, 20.79, 20.87, 20.95, 21.07, 21.13 (7 q, 7 COCH_3); 41.1 (t, CH_2N), 61.61 (t, C-6'), 62.8 (t, C-6), 66.9 (t, CH_2Ph), 68.1 (d, C-5'), 68.7 (d, C-3'), 69.5 (t, OCH_2), 69.8 (d, C-2'), 70.1 (d, C-2), 72.2 (d, C-5), 72.4 (d, C-4), 72.6 (d, C-4'), 75.4 (d, C-3), 95.7 (d, C-1'), 100.8 (d, C-1), 128.4, 128.7 (2 d, *o*-, *m*-, *p*-*C* from *C*$_6$H$_5$), 136.6 (s, *i*-*C* from *C*$_6$H$_5$), 156.5 (s, N*C*OO), 169.6, 169.9, 170.1, 170.4, 170.65, 170.72 (6 s, 7 *C*OCH_3); IR: $\tilde{\nu}$ (cm^{-1}) = 3394, 2959, 1752, 1523, 1432, 1369, 1238, 1040; HRMS (ESI+): *m/z* calcd for $C_{36}H_{47}NO_{20}$ $[M+H]^+$ 814.2770, found 814.2772.

2-Aminoethyl α-D-glucopyranosyl-(1→4)-β-D-glucopyranoside (8)

The glycosylated maltose **18** (304 mg, 0.374 mmol) was first deacetylated according to General Procedure 4 followed by hydrogenation as described in General Procedure 5. The free maltoside **8** (144 mg, 0.372, 99 %) was obtained as a white solid.

m.p. = 165–167 °C; $[\alpha]_D^{20}$ = +69.7 (*c* 6.4, MeOH); IR: $\tilde{\nu}$ (cm^{-1}) = 3346, 1643, 1039; HRMS (ESI+): *m/z* calcd for $C_{14}H_{27}NO_{11}$ $[M+H]^+$ 386.1662, found 386.1662.

Synthesis of aminoethyl Neu5Ac (9)

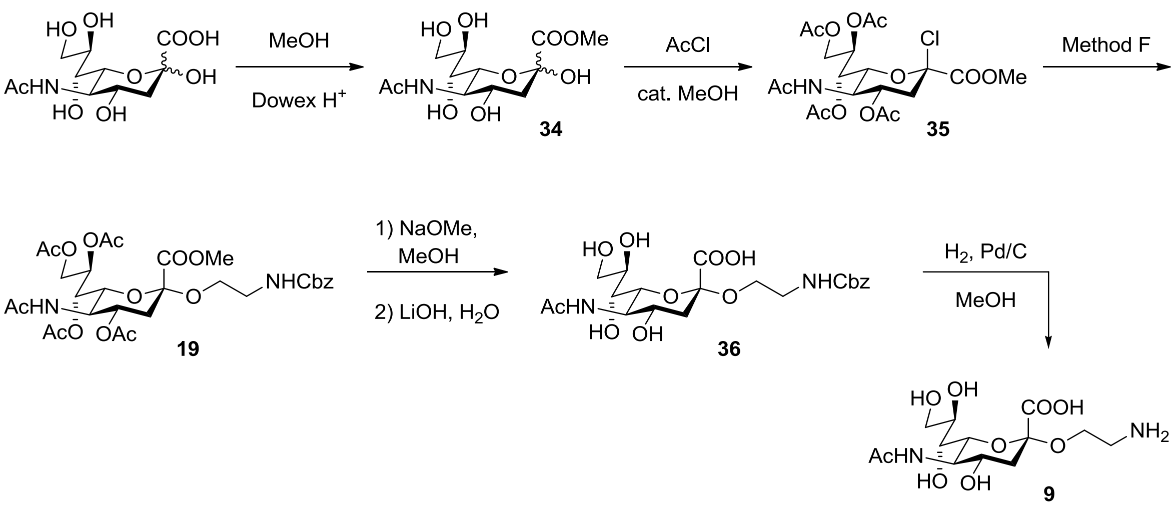

N-Acetyl β-neuraminic acid methyl ester (34)

N-Acetyl neuraminic acid (Codexis, 2.00 g, 6.47 mmol) was stirred with Dowex 50WX4 (500 mg) in dry methanol (150 mL) overnight at r.t. The removal of the resin by filtration and evaporation of MeOH *in vacuo* afforded methyl ester **34** (2.0 g, 6.19 mmol, 96 %) as a white solid.

m.p. = 179–180 °C (dec.), Lit. [8] m.p. = 180–182 °C (dec.) $[\alpha]_D^{20}$ = −24.1 (*c* 1.2, MeOH), Lit. [9] $[\alpha]_D^{20}$ = −28 (*c* 3.5, H_2O), Lit. [8] $[\alpha]_D^{20}$ = −28 (*c* 1, H_2O); ^{1}H NMR (400 MHz, D_2O): δ (ppm) = 1.92 (dd, *J* = 11.7, 12.9 Hz, 1H, 3-H_a), 2.06 (s, 3H, COCH_3), 2.32 (dd, *J* = 4.9, 13.0 Hz, 1H, 3.55 (dd, *J* = 0.9, 9.2 Hz, 1H, 7-H), 3-H_e), 3.62 (dd, *J* = 6.3, 11.8 Hz, 1H, 9-H_a), 3.74 (ddd, *J* = 2.5, 6.3, 9.2 Hz, 1H, 8-H), 3.842 (dd, *J* = 2.5, 11.7 Hz, 1H, 9-H_b), 3.843 (s, 3H, COOCH_3), 3.93 (dd, *J* = 10.1, 10.6 Hz, 1H, 5-H), 4.07 (ddd, *J* = 4.9, 10.1, 11.6 Hz, 1H, 4-H), 4.08 (ddd, *J* = 1.0, 10.5 Hz, 1H, 6-H); ^{13}C NMR (101 MHz, D_2O) δ (ppm) = 25.0 (q, COCH_3), 41.6 (t, C-3), 55.0 (d, C-5), 56.4 (q, COOCH_3), 66.1 (t, C-9), 69.6 (d, 4), 71.1 (d, 7), 73.0 (d, 8), 73.2 (d, 6), 98.2 (s, C-2), 174.3, 177.7 (2s, 2 *C*O); IR: $\tilde{\nu}$ (cm^{-1}) = 3305, 1740, 1640, 1556, 1309, 1280, 1130, 1068, 1036; HRMS (ESI+): *m/z* calcd for $C_{12}H_{21}NO_9$ $[M+Na]^+$ 346.1114, found 346.1117.

Methyl 5-acetamido-4,7,8,9-tetra-*O*-acetyl-2,3,5-trideoxy-2-chloro-D-*glycero*-β-D-*galacto*-2-nonulopyranosonate (35)

Methyl ester **34** (2.00 g, 6.19 mmol) was dissolved in acetyl chloride (60 mL) and abs methanol (1.2 mL) added. The reaction vessel was sealed and the mixture stirred at r.t. for 5 d. Evaporation to dryness following by short column chromatography on silica (EtOAc/hexane 80/20) gave chloride **35** as a yellowish solid. Recrystallisation from diethyl ether/petroleum ether gave the title compound as a white crystalline solid (2.28 g, 4.47 mmol, 72 %).

$[\alpha]_D^{20}$ = −165 (*c* 0.53, CH_2Cl_2), Lit. [9] $[\alpha]_D^{20}$ = −61 (*c* 1, $CHCl_3$), Lit. [8] $[\alpha]_D^{20}$ = −68 (*c* 1, $CHCl_3$); ^{1}H NMR (500 MHz, $CDCl_3$): δ (ppm) = 1.89 (s, 3H, $COCH_3$), 2.04 (s, 3H, COCH_3), 2.04 (s, 3H, COCH_3), 2.06 (s, 3H, COCH_3), 2.11 (s, 3H, COCH_3), 2.25 (dd, *J* = 11.3, 13.9 Hz, 1H, 3-H_a), 2.77 (dd, *J* = 4.8, 13.9 Hz, 1H, 3-H_e), 3.86 (s, 3H, COOCH_3), 4.05 (dd, *J* = 6.0, 12.5 Hz, 1H, 9-H_a), 4.20 (q, *J* = 10.4 Hz, 1H, 5-H), 4.35 (dd, *J* = 2.4, 10.8 Hz, 1H, 6-H), 4.42 (dd, *J* = 2.6, 12.5 Hz, 1H, 9-H_b), 5.15 (ddd, *J* = 2.6, 6.0, 6.8 Hz, 1H, 8-H), 5.38 (ddd, *J* = 4.8, 10.8, 11.2 Hz, 1H, 4-H), 5.46 (dd, *J* = 2.4, 6.8 Hz, 1H, 7-H), 5.69 (d, *J* = 10.1 Hz,

1H, NH); ^{13}C NMR (126 MHz, $CDCl_3$) δ (ppm) = 20.86, 20.90, 21.0, 21.1, 23.2 (5 q, 6 $COCH_3$), 40.7 (t, C-3), 48.7 (d, C-5), 53.9 (q, COO*C*H_3), 62.2 (t, C-9), 67.0 (d, C-7), 68.8 (d, C-4), 70.1 (d, C-8), 74.0 (d, C-6), 96.7 (s, C-2), 165.7, 169.9, 170.1, 170.5, 170.8, 171.1 (6 s, 6 CO); IR: $\tilde{\nu}$ (cm^{-1}) = 1743, 1370, 1222, 1036; HRMS (ESI+): *m/z* calcd for $C_{20}H_{28}ClNO_{12}$ $[M\text{-}Cl+H_2O]^+$ 492.1717, found 492.1716.

Methyl 5-acetamido -2-*O*-(2-benzyloxycarbonylaminoethyl)-4,7,8,9-tetra-*O*-acetyl-3,5-dideoxy-D-*glycero*- α-D-*galacto*-2-nonulopyranosonate (19)

Sialyl chloride **35** (400 mg, 0.784 mmol) and *N*-Cbz-aminoethanol (383 mg, 1.96 mmol, 2.5 equiv) were dissolved in CH_2Cl_2 (6 mL) and 500 mg of 4 Å molecular sieves was added. After stirring for 1 h, Ag_2CO_3 (433 mg, 1.57 mmol, 2 equiv) was added and the mixture stirred for 16 h at room temperature with exclusion of light. The solid was filtered through Celite, washed with CH_2Cl_2 and the filtrate evaporated to dryness. Column chromatography (EtOAc/hexane 80:20) gave 368 mg (0.55 mmol, 70 %) of glycoside **19** as a colourless foam. $[\alpha]_D^{20}$ = +162 (*c* 1.13, CH_2Cl_2); ^{1}H NMR (400 MHz, $CDCl_3$): δ (ppm) = 1.88 (s, 3H, NCOC*H*$_3$), 1.93 (dd, *J* = 12.2, 13.0 Hz, 1H, 3-H_a), 2.03 (s, 3H, OCOC*H*$_3$), 2.04 (s, 3H, OCOC*H*$_3$), 2.05 (s, 3H, OCOC*H*$_3$), 2.14 (s, 3H, OCOC*H*$_3$), 2.56 (dd, *J* = 4.6, 13.0 Hz, 1H, 3-H_e), 3.30–3.47 (m, 3H, OC*H*$_a$H$_b$C*H*$_2$N), 3.76 (s, 3H, OCH_3), 3.80 (m, 1H, OCH$_a$*H*$_b$CH$_2$N), 4.01–4.11 (m, 2H, 5-H, 9-H_a), 4.16 (dd, *J* = 2.1, 10.7 Hz, 1H, 6-H), 4.27 (dd, *J* = 2.4, 12.4 Hz, 1H, 9-H_b), 4.85 (ddd, *J* = 4.6, 10.3, 12.2 Hz, 1H, 4-H), 5.11 (s, 2H, C*H*$_2$Ph), 5.26 (d, *J* = 10.0 Hz, 1H, NH), 5.30 (dd, *J* = 2.1, 8.3 Hz, 1H, 7-H), 5.37 (ddd, *J* = 2.4, 5.7, 8.3 Hz, 1H, 8-H), 7.25–7.39 (m, 5H, C$_6$*H*$_5$); ^{13}C NMR (101 MHz, $CDCl_3$) δ (ppm) = 20.7, 20.8, 20.9, 21.1 (4 q, 4 OCO*C*H$_3$), 23.2 (q, NHCO*C*H$_3$), 37.9 (t, C-3), 40.9 (t, OCH$_2$*C*H$_2$N), 49.4 (d, C-5), 52.9 (q OCH_3), 62.4 (t, C-9), 64.2 (t, O*C*H$_2$CH$_2$N), 66.7 (t, O*C*H$_2$Ph), 67.2 (d, C-7), 68.4 (d, C-8), 68.9 (d, C-4), 72.6 (d, C-6), 98.7 (s, C-2), 128.18, 128.20, 128.6 (3 d, *o*-, *m*-, *p*-C from C_6H_5), 136.5 (s, *i*-C from C_6H_5), 156.3 (s, NH*C*OO), 168.4 (s, NH*C*OCH$_3$), 170.0, 170.1, 170.3, 170.7, 171.0 (5 s, 4 *C*OCH$_3$, *C*OOCH$_3$); IR: $\tilde{\nu}$ (cm^{-1}) = 1742, 1662, 1540, 1445, 1371, 1223, 1127, 1038, 736; HRMS (ESI+): *m/z* calcd for $C_{30}H_{40}N_2O_{15}$ $[M+H]^+$ 669.2507, found 669.2518.

5-Acetamido -2-*O*-(2-benzyloxycarbonylaminoethyl)-3,5-dideoxy-D-*glycero*-D-*galacto*-2-nonulopyranosidonic acid (36)

Aminoethyl glycoside **19** (80 mg, 0.12 mmol) was dissolved in methanol (3 mL) and sodium methoxide (6.4 mg, 0.12 mmol), dissolved in 1 mL methanol added. After stirring at r.t. for 6 h, the solution was neutralised with Dowex 50WX8-100 (H^+) resin, filtered and concentrated *in vacuo*. The residue was dissolved in 3 mL water/methanol (4:1), treated with LiOH (8.6 mg, 0.36 mmol) and the solution was stirred overnight at room temperature. After neutralisation with Dowex 50WX8-100, the resin was filtered off and washed with methanol/water (1:1). The filtrate was concentrated in vacuum to remove most of the methanol and then freeze dried to yield the free glycoside **36** as a white solid (52 mg, 0.11 mmol, 89 %).

$[\alpha]_D^{20}$ = −42.5 (*c* 0.4, MeOH); ^{1}H NMR (400 MHz, MeOD): δ (ppm) = 1.68 (dd, *J* = 11.4, 12.5 Hz, 1H, 3-Ha), 1.97 (2, 3H, COCH_3), 2.71 (dd, *J* = 4.5, 12.7 Hz, 1H, 3-H_e), 3.24–3.27 (m, 2H, CH_2NH), 3.46–3.51 (m, 2H, 7-H, CH_aH_bCH$_2$NH), 3.56 (bd, *J* = 10.7 Hz, 1H, 6-H), 3.59 (dd, *J* = 6.0, 11.8 Hz, 1H, 9-H_b), 3.65–3.74 (m, 2H, 4-H, 5-H), 3.75–3.83 (m, 3H, 8-H. 9-H_b, CH$_a$$H_bCH_2$NH), 5.04 (s, 2H, C$H_2$Ph), 7.23–7.34 (m, 5H, C$_6$$H_5$); ^{13}C NMR (101 MHz, MeOD) δ (ppm) = 175.4, 172.5 (2 s, 2 CO), 158.9 (s, N*C*OO), 138.4 (s, *i*-C from C_6H$_5$), 129.5, 128.9, 128.8 (3 d, *o*-, *m*-, *p*-C from C_6H$_5$), 100.5 (s, C-2), 74.8 (C-6), 72.8 (d, C-8), 70.1 (d, C-7), 68.9 (d, C-4), 67.4 (t, *C*H$_2$Ph), 64.5, 64.2 (2 t, C-9, *C*H$_2$CH$_2$NH), 53.9 (d, C-5), 42.0, 41.9 (2 t, C-3, *C*H$_2$NH), 22.6 (q, CO*C*H$_3$); IR: $\tilde{\nu}$ (cm^{-1}) = 3310, 2949, 1698, 1640, 1554,1535, 1456, 1372, 1273, 1199, 1131, 1073, 1031, 900; HRMS (ESI+): *m/z* calcd for $C_{21}H_{30}N_2O_{11}$ $[M+Na]^+$ 509.1747, found 509.1752.

5-Acetamido-2-*O*-(2-aminoethyl)-3,5-dideoxy-D-*glycero*-α-D-*galacto*-2-nonulopyranosidonic acid (9)

Following the General Procedure 5, the sialic acid **36** (52 mg, 0.11 mmol) was hydrogenated in methanol (5 mL) with palladium on carbon. After 6 h solution was filtered through Celite to remove the catalyst, the filter cake washed with methanol and the filtrates concentrated *in vacuo*. The product was redissolved in water, treated with activated charcoal and filtered. Lyophilisation of the filtrate gave the deprotected sialic acid **9** (36 mg, 0.10 mmol, 96 %) as a white solid.

$[\alpha]_D^{20}$ = −35 (*c* 0.2, MeOH), Lit. [10] $[\alpha]_D^{20}$ = −17 (*c* 0.5, H_2O), Lit. [9] $[\alpha]_D^{20}$ = −18.5 (*c* 1, H_2O); ^{1}H NMR (400 MHz, D_2O): δ (ppm) = 1.69 (t, *J* = 12.2 Hz, 1H, 3-H_a), 2.00 (s, 3H,

$COCH_3$), 2.69 (dd, *J* = 4.7, 12.5 Hz, 1H, 3-H_e), 3.15 (t, *J* = 5.1 Hz, 2H, $NHCH_2$), 3.54–3.69 (m, 5H), 3.77–3.84 (m, 3H), 3.89–3.96 (m, 1H, 8-H); ^{13}C NMR (101 MHz, D_2O) δ (ppm) = 21.9 (q, $COCH_3$), 39.3 (t), 39.7 (t), 51.8 (d), 60.3 (t), 62.6 (t), 68.0 (d), 68.1 (d), 71.6 (d), 72.6 (d), 100.4 (s, C-2), 173.4, 175.1 (2 s, 2 CO); IR: $\tilde{\nu}$ (cm^{-1}) = 3300, 3086, 2939, 1614, 1435, 1388, 1378, 1115, 1073, 1031; HRMS (ESI+): *m/z* calcd for $C_{13}H_{24}N_2O_9$ $[M+Na]^+$ 375.1380, found 375.1378.

References

1. Hudson, C. S.; Johnson, J. M. *J. Am. Chem. Soc.* **1915,** *37,* 2748–2753. doi:10.1021/ja02177a023
2. Schroeder, L. R.; Counts, K. M.; Haigh, F. C. *Carbohydr. Res.* **1974,** *37,* 368–372. doi:10.1016/S0008-6215(00)82927-4
3. Orlandi, S.; Annunziata, R.; Benaglia, M.; Cozzia, F.; Manzoni, L. *Tetrahedron* **2005,** *61,* 10048–10060. doi:10.1016/j.tet.2005.08.018
4. Denoyelle, S.; Polidori, A.; Brunelle, M.; Vuillaume, P. Y.; Laurent, S.; ElAzhary, Y.; Pucci, B. *New J. Chem.* **2006,** *30,* 629–646. doi:10.1039/b513944a
5. Susaki, H.; Suzuki, K.; Ikeda, M.; Yamada, H.; Watanabe, H. K. *Chem. Pharm. Bull.* **1994,** *42,* 2090–2096.
6. Kamst, E.; Zegelaar-Jaarsveld, K.; van der Marel, G. A.; van Boom, J. H.; Lugtenberg, B. J. J.; Spaink, H. P. *Carbohydr. Res.* **1999,** *321,* 176–189. doi:10.1016/S0008-6215(99)00190-1
7. King, R. R.; Cooper, F. P.; Bishop, C. T. *Carbohydr. Res.* **1977,** *55,* 83–93. doi:10.1016/S0008-6215(00)84445-6
8. Ogura, H.; Furuhata, K.; Itoh, M.; Shitori, Y. *Carbohydr. Res.* **1986,** *158,* 37–51. doi:10.1016/0008-6215(86)84004-6
9. Roy, R.; Laferriere, C. A. *Can. J. Chem.* **1990,** *68,* 2045–2054. doi:10.1139/v90-313

10. Eschenfelder, V.; Brossmer, R. *Carbohydr. Res.* **1980,** *78*, 190–194. doi:10.1016/S0008-6215(00)83672-1
